# Identification and Verification of Necroptosis-Related Gene Signature With Prognosis and Tumor Immune Microenvironment in Ovarian Cancer

**DOI:** 10.3389/fimmu.2022.894718

**Published:** 2022-06-24

**Authors:** Zitao Wang, Ganhong Chen, Fangfang Dai, Shiyi Liu, Wei Hu, Yanxiang Cheng

**Affiliations:** ^1^ Department of Obstetrics and Gynecology, Renmin Hospital of Wuhan University, Wuhan, China; ^2^ Department of Pathology, The People's Hospital of Honghu, Honghu, Hubei, China; ^3^ Department of Obstetrics and Gynecology Ultrasound, Renmin Hospital of Wuhan University, Wuhan, China

**Keywords:** ovarian cancer, necroptosis, signature, tumor immune microenvironment, immunotherapy, prognosis

## Abstract

Ovarian cancer is the most lethal heterogeneous disease among gynecological tumors with a poor prognosis. Necroptosis, the most studied way of death in recent years, is different from apoptosis and pyroptosis. It is a kind of regulated programmed cell death and has been shown to be closely related to a variety of tumors. However, the expression and prognosis of necroptosis-related genes in ovarian cancer are still unclear. Our study therefore firstly identified the expression profiles of necroptosis-related genes in normal and ovarian cancer tissues. Next, based on differentially expressed necroptosis-related genes, we clustered ovarian cancer patients into two subtypes and performed survival analysis. Subsequently, we constructed a risk model consisting of 5 genes by LASSO regression analysis based on the differentially expressed genes in the two subtypes, and confirmed the strong prognostic ability of the model and its potential as an independent risk factor *via* survival analysis and independent risk factor analysis. Based on this risk model, patients were divided into high and low risk groups. By exploring differentially expressed genes, enrichment functions and tumor immune microenvironment in patients in high and low risk groups, the results showed that patients in the low risk group were significantly enriched in immune signaling pathways. Besides, immune cells content, immune function activity was significantly better than the high-risk group. Eventually, we also investigated the sensitivity of patients with different risk groups to ICB immunotherapy and chemotherapy drugs. In conclusion, the risk model could effectively predict the survival and prognosis of patients, and explore the tumor microenvironment status of ovarian cancer patients to a certain extent, and provide promising and novel molecular markers for clinical diagnosis, individualized treatment and immunotherapy of patients.

## Introduction

Ovarian cancer (OC) is the deadliest malignancy of gynecological tumors, causing approximately 150,000 female deaths each year (1). Besides, owing to its’ heterogeneity, complex and uncertain etiology and lack of typical clinical symptoms in the early stage, 75% of OC patients are diagnosed as an advanced stage, and more than 70% of patients recurred after treatment (2). Therefore, the prognosis largely depends on the clinical stage and early prevention. In the past few years, the advance of the diagnosis, surgery and targeted therapy have largely improved the survival, but the lack of effective indicators of occurrence and recurrence is still the main obstacle at present, so the identification of reliable prognostic biomarkers are indispensable for prolonging the overall survival of OC patients. At present, CA-125 and human epididymal protein 4 are the most commonly used predictive markers in clinical practice (3). Nevertheless, due to the complex molecular mechanisms that affect ovarian cancer prognosis, single-gene prediction models tend to be less accurate and sensitive, while multiple genes-based models tend to show better results in predicting the prognosis of various tumors.

Necroptosis, a regulated programmed cell death, is mechanistically similar to apoptosis and morphologically similar to necrosis, whose key regulators of necroptosis include receptor interacting protein kinase 1, and mixed lineage kinase domains such as pseudokinase, together forming a necrosome complex (4, 5), thus activating programmed necrosis through TNF receptor superfamily, T cell receptors, Toll-like receptors and etc (6). There is increasing evidence that necroptosis is involved in the pathogenesis of many diseases, such as Parkinson’s disease, infectious diseases and cancers (7, 8). In non-Hodgkin’s lymphoma, single nucleotide polymorphisms in the RIP3 gene were detected in 458 patients and were associated with an increased risk of non-Hodgkin’s lymphoma, suggesting that inheritance of the RIP3 gene variations might contribute to the onset of the disease (9). Several studies have even found that necroptosis regulators might be prognostic biomarkers for cancer and certain diseases (10, 11). In pancreatic cancer, necroptosis could promote tumor cell migration and invasion by releasing CXCL5 (12). Besides, Najafov et al. revealed that necroptosis could promote tumor metastasis and T cell death (13). Interestingly, necroptosis, an alternative mode of programmed cell death to overcome apoptosis resistance, might trigger and amplify the application of antitumor immunity in cancer therapy. However, the role of necroptosis in the prognosis and underlying molecular mechanisms of OC is currently unclear.

## Materials and Methods

### Publicly Attainable Expression Datasets

We obtained the RNA-seq data, mutation data and related clinical information of normal ovarian tissue and ovarian cancer tissue from GTEX (The Genotype-Tissue Expression, https://xenabrowser.net/datapages/) and TCGA (The Cancer Genome Atlas, https://portal.gdc.cancer.gov/repository), and then normalize the expression profile data to remove batch effects for further analysis. The RNA-seq data and clinical information in external validation cohort were downloaded from the GEO (Gene Expression Omnibus, https://www.ncbi.nlm.nih.gov/geo/, ID: GSE32062) were displayed in **Table 1**. All analyses were performed with R 4.0.1.

**Table 1:** Clinical characteristics of the samples in TCGA and GEO cohort.

	TCGA(n=587)	GEO(n=260)
**Age, years (range)**	59 (26,89)	64 (2,96)
**Grade**		
Grade 1	6 (1.0%)	0 (0.0%)
Grade 2	69 (11.8%)	131 (50.1%)
Grade 3	495 (84.3%)	129 (49.9%)
Grade 4	1 (0.2%)	0 (0.0%)
NA	16 (2.7%)	0 (0.0%)
**Stage**		
Stage III	NA	204 (78.5%)
Stage IV	NA	56 (21.5%)
**Recurrence** positive negative	NA NA	67 (25.8%) 193 (74.2%)
**Vital status**		
Alive	282 (48.0%)	139 (53.5%)
Deceased	305 (52.0%)	121 (46.5%)

NA, Not applicable.

### Identification of Differentially Expressed Necroptosis-Related Genes

By searching the previous literature, we obtained 75 necroptosis genes in **Table 2**. Then we obtained the expression profiles of 75 necroptotic genes, and obtained 33 differentially expressed necroptosis-related genes through the limma package (Log2 fold change < 1, false discovery rate (FDR) < 0.05, and *P* < 0.05). STRING and correlation analysis were used to explore the interaction network of these differentially expressed genes (DEGs) (cutoff = 0.2). Furthermore, we performed function analyses to investigate the enriched functions and pathways of the DEGs.

**Table 2:** Necroptosis-related genes were presented.

Genes	Full-names
ALDH2	aldehyde dehydrogenase 2 family member
CXCL1	C-X-C motif chemokine ligand 1
EZH2	enhancer of zeste 2 polycomb repressive complex 2 subunit
HMGB1	high mobility group box 1
NDRG2	NDRG family member 2
NR2C2	nuclear receptor subfamily 2 group C member 2
PGAM5	PGAM family member 5, mitochondrial serine/threonine protein phosphatase
TLR2	toll like receptor 2
TLR4	toll like receptor 4
ALK	ALK receptor tyrosine kinase
APP	amyloid beta precursor protein
ATRX	ATRX chromatin remodeler
AXL	AXL receptor tyrosine kinase
BACH2	BTB domain and CNC homolog 2
BCL2	BCL2 apoptosis regulator
BCL2L11	BCL2 like 11
BNIP3	BCL2 interacting protein 3
BRAF	B-Raf proto-oncogene, serine/threonine kinase
CASP8	caspase 8
CD40	CD40 molecule
CDKN2A	cyclin dependent kinase inhibitor 2A
CFLAR	CASP8 and FADD like apoptosis regulator
CYLD	CYLD lysine 63 deubiquitinase
DDX58	DExD/H-box helicase 58
DIABLO	diablo IAP-binding mitochondrial protein
DNMT1	DNA methyltransferase 1
EGFR	epidermal growth factor receptor
FADD	Fas associated *via* death domain
FAS	Fas cell surface death receptor
FASLG	Fas ligand
FLT3	fms related receptor tyrosine kinase 3
GATA3	GATA binding protein 3
HAT1	histone acetyltransferase 1
HDAC9	histone deacetylase 9
HSP90AA1	heat shock protein 90 alpha family class A member 1
HSPA4	heat shock protein family A (Hsp70) member 4
ID1	inhibitor of DNA binding 1, HLH protein
IDH1	isocitrate dehydrogenase [NADP(+)] 1
IDH2	isocitrate dehydrogenase [NADP(+)] 2
IPMK	inositol polyphosphate multikinase
ITPK1	inositol-tetrakisphosphate 1-kinase
KLF9	Kruppel like factor 9
LEF1	lymphoid enhancer binding factor 1
MAP3K7	mitogen-activated protein kinase kinase kinase 7
MAPK8	mitogen-activated protein kinase 8
MLKL	mixed lineage kinase domain like pseudokinase
MPG	N-methylpurine DNA glycosylase
MYC	MYC proto-oncogene, bHLH transcription factor
MYCN	MYCN proto-oncogene, bHLH transcription factor
OTULIN	OTU deubiquitinase with linear linkage specificity
PANX1	pannexin 1
PLK1	polo like kinase 1
RIPK1	receptor interacting serine/threonine kinase 1
RIPK3	receptor interacting serine/threonine kinase 3
RNF31	ring finger protein 31
SIRT1	sirtuin 1
SIRT2	sirtuin 2
SIRT3	sirtuin 3
SLC39A7	solute carrier family 39 member 7
SPATA2	spermatogenesis associated 2
SQSTM1	sequestosome 1
STAT3	ignal transducer and activator of transcription 3
STUB1	STIP1 homology and U-box containing protein 1
TARDBP	TAR DNA binding protein
TERT	telomerase reverse transcriptase
TLR3	toll like receptor 3
TNF	tumor necrosis factor
TNFRSF1A	TNF receptor superfamily member 1A
TNFRSF1B	TNF receptor superfamily member 1B
TNFRSF21	TNF receptor superfamily member 21
TNFSF10	TNF superfamily member 10
TRAF2	TNF receptor associated factor 2
TRIM11	tripartite motif containing 11
TSC1	TSC complex subunit 1
USP22	ubiquitin specific peptidase 22

### NMF Consensus Clustering

NMF clustering was performed to identify stable sample clusters based on 50 iterations according to the Brunet method using genes associated with energy metabolism. In addition, the cluster number, represented by k, was set as 2–10, whilst the best cluster number was calculated based on the cluster cophenetic correlation and the observed consensus map. The mean silhouette width of the consensus membership matrix was determined using the “NMF” function in the R package.

### Development and Validation of the Necroptosis‐Related Prognostic Signature for Ovarian Cancer

Based on the obtained clinical information of ovarian cancer, univariate Cox analysis was used to identify prognosis-related genes as the Cox *P* value of 0.05, and we eventually obtained 7 genes closely associated with prognosis. To further construct the risk model, the “glmnet” package was used to perform LASSO regression analysis using 10-fold cross-validation and a *P*-value of 0.05, and 1,000 cycles’ running. For each cycle, 1000 random stimuli were set to prevent overfitting. Ultimately, a 5-gene model was successfully constructed, and the risk score formula is as follows: Risk Score= ∑ Xi × Yi (X: coefficients, Y: gene expression level). Based on this formula, patients in the TCGA database were divided into high-risk and low-risk groups according to the median score, and each patient’s risk score and survival status were presented in the form of a heatmap, while Rtsne and stats were used for dimensionality reduction analysis to distinguish the patients between high and low risk groups. Kaplan-Meier analysis was utilized to identify the differences in overall survival (OS) between high and low risk groups. Finally, in order to evaluate the predictive power of the risk model, the “survival”, “survminer” and “timeROC” packages calculated the area under curve (AUC) values ​​for 1, 3, and 5 years. Besides, the GEO cohort was used to validate the risk model. Patients in the validation set were also divided into high and low risk groups by applying the median risk scores, and PCA, ROC, and Kaplan-Meier (KM) analyses were also applied to the GEO dataset.

### Independent Prognostic Analysis of the Signature

Before the analysis, we screened and grouped for clinical characteristics. Owing to the differences in age between patients, we used age = 65 as the distinguishing criterion according to the screening criteria of previous researches. Besides, we divided Grade 1-2 into one group, and Grade 3-4 into another group to better compare survival analysis results. To investigate whether risk score could be an independent risk factor for ovarian cancer patients, univariate and multivariate COX analyses were used to investigate clinical traits such as age, grade and risk score, and the results were presented in the form of forest plots.

### Construction of the Nomogram

We integrated clinical characteristics with risk scores and applied the rms R package to construct the nomogram predicting 1, 3, 5-year survival probabilities of patients, and a calibration curve to verify predictive power.

### Comparison Between the Signature and the Other Established Signature

Based on the model constructed in this study, we compared the risk models that have been constructed in previous studies, and identified the predictive ability of the model by calculating the ROC value of different models, the *P* value of survival analysis, and the C-index value of the model.

### Functional Enrichment Analysis Between High and Low Risk Groups

Patients in the TCGA cohort were divided into high and low risk groups according to the median risk score, and the limma package was used to identify differentially expressed genes (|log2FC| ≥ 1 and FDR < 0.05). Based on these differential genes, Gene Ontology (GO) and Kyoto Encyclopedia of Genes and Genomes (KEGG) functional enrichment analyses were performed.

### The Mutation Spectrum of Patients in TCGA

The somatic mutation information of patients was downloaded from TCGA, and the frequency of somatic mutation of each patient and the mutation frequency of different genes were calculated by the maftools package. Meanwhile, based on the risk model, the mutation spectrum of patients in the high and low risk groups was estimated to compare whether there existed significant difference between the groups. In addition, the calculated TMB scores were compared to explore the difference and the relationship in the high and low risk groups.

### Analysis of Copy Number Variation

Copy-number variant (CNV) refers to copy number variation, which greatly enriches the diversity of genetic variation in the genome. GISTIC software was used to identify genes exhibiting significant amplification or deletion.

### Tumor Immune Microenvironment

In order to estimate the immune infiltration of ovarian cancer patients, the CIBERSORT program was used, and the R package “Cibersort” was used to obtain the normalized enrichment score of 22 immune cells in the high and low risk groups, and the KM survival analysis of high and low immune infiltrating cells and functional patient survival prognosis. Other than that, ssGSEA was conducted to calculate the scores of infiltrating immune cells and to evaluate the activity of immune-related pathways *via* gsva package. Except for that, the correlation between the signature and immune cell markers was investigated. Cell markers were chosen according to CellMarker database

### Sensitivity to Chemotherapy

To explore the correlation between risk models and chemotherapy in ovarian cancer patients, the pRRophetic package was used to calculate the sensitivity of patients in high and low risk groups to multiple chemotherapeutic agents.

### Evaluation of the Immunotherapy Between Groups

The expression of immune checkpoint molecules was compared between high and low risk groups, and we explored whether there was a significant difference between high and low risk patients on ICB treatment.

### Risk Correlation Analysis

The content of immune cells and immune genes in each sample was integrated, and the association between immune genes, immune cells, and TMB and risk scores was investigated *via* Pearson correlation analysis, visualized by the corrplot package.

### The Expression of Key Genes in Clinical Samples

Adjacent non-tumor and ovarian cancer tissues were obtained from the Gynecology and Obstetrics in Renmin Hospital of Wuhan University. All the patients provided informed consent and were approved by the Ethics Committee of Renmin Hospital of Wuhan University to collect 9 cases of OC tissues and corresponding paracancerous tissues. The clinical parameters were displayed in **Table 3**. Total RNA from ovarian cancer and paracancerous tissue samples were extracted and quantitated by qRT-PCR, where GAPDH was used as an internal control.

**Table 3:** Clinicopathological parameters of patients.

Case id	Age	Gender	Tumor size(cm)	TNM	Histological type	Chemotherapy	Radiotherapy
1	58	Female	1*1*1	T3N1M0	High-grade Serous IIIC	Paclitaxel; Lobaplatin	NA
2	53	Female	3*2.5*2.5	T3N1M1	High-grade Serous IVB	Taxol; Carboplatin	NA
3	68	Female	5*3*2.2	T3N1M0	High-grade Serous IIIC	nab-paclitaxel, carboplatin	NA
4	63	Female	15*13*13	T3N1M0	High-grade Serous IIIC	Lobaplatin	NA
5	65	Female	16.5*11.5*2.5	T3N1M0	High-grade Serous IIIC	Cisplatin	NA
6	45	Female	11.15*8.20*9.22	T3N1M0	High-grade Serous IIIC	Cisplatin	NA
7	53	Female	11*9.5	T3N1M1	High-grade Serous IIIC	Taxol; Carboplatin	NA
8	45	Female	8*5*1.2	T3N0M0	High-grade Serous IIIB	Taxol; Carboplatin	NA
9	44	Female	4*4*3	T3N1M1	High-grade Serous IVA	Taxol; Carboplatin	NA

NA, Not applicable.

## Results

### Identification of Differentially Expressed Necroptosis-Related Genes

We integrated normal tissue samples in GTEX and tumor tissue samples in TCGA to obtain the expression profiles of 75 necroptosis-related genes, and then identified 33 DEGs (LogFC>1, FDR<0.05). *NDRG2*, *ALDH2*, *DIABLO*, *TARDBP*, *KLF9*, *HMGB1*, *CFLAR*, *FAS*, *SQSTM1*, *SIRT2*, *RNF31*, *SIRT3*, *BCL2*, *CYLD* and *TSC1* were downregulated in tumor tissues, while *HSPA4*, *CA*SP8, *PANX1*, *RIPK1*, *FADD*, *LEF1*, *SPATA2*, *EZH2*, *TNFSF10*, *DDX58*, *MYCN*, *TNF*, *PGAM5*, *CXCL1*, *PLK1*, *TNFRSF21*, *IDH2* and *CDKN2A* were upregulated (**Supplementary Figure 1A**). To further investigate the interaction between these genes, we performed a PPI analysis using STRING (the highest confidence=0.4) in **Supplementary Figure 1B**, while the correlation network is presented in **Supplementary Figure 1C**, which indicated that *CASP8*, *CDKN2A*, *CFLAR*, *CYLD*, *DDX58* and *DIABLO* were hub genes, playing a central role in the tumorigenesis and development of OC. Furthermore, we performed the function analyses to investigate the enriched functions and pathways and the results showed that necroptotic process, negative regulation of reactive oxygen species and NF−kappa B signaling pathway were enriched in the KEGG terms (**Supplementary Figure 1D**), negative regulation of reactive oxygen species, tumor necrosis factor receptor superfamily binding and tumor necrosis factor receptor superfamily binding in the GO terms (**Supplementary Figure 1E**).

### NMF-Based Sample Classification

**Figure 1:**
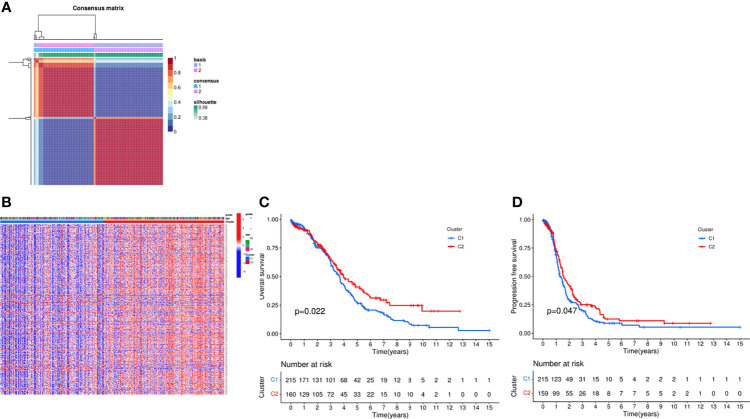
Consensus clustering analysis of the patients *via* NMF algorithm. **(A)** NMF clustering using necroptosis-related genes. Patients were divided into cluster 1 and cluster 2. **(B)** Heat map of two clusters defined by the necroptosis -related genes expression profile. **(C)** Kaplan-Meier curve between two clusters via survival analysis in OS. **(D)** Kaplan-Meier curve between two clusters via survival analysis in PFS.

In order to explore the relationship between DEGs and ovarian cancer subtypes, we performed NMF clustering on 379 patients in TCGA. By continuously running the NMF function and extracting the cophenetic coefficient, we obtained the best rank value, that is, the number of clusters = 2 (**Figure 1A**), and we could find that patients were well divided into two groups, and heatmaps of gene expression and clinical characteristics were presented in **Figure 1B**. At the same time, we investigated whether there is a difference in survival between the two groups, and found that there were significant differences in OS and progression free survival (PFS), and the survival prognosis of patients in the C2 group was significantly better than that in the C1 group (*P* = 0.022, 0.047) (**Figures 1C, D**). Random forest is a decision tree-based machine learning algorithm that can be used for sample classification or regression tasks, thus exploring complex non-linear interdependencies between variables to distinguish the key components between the two groups. *Via* the “randomForest” R package, we figured out several key genes involved in the occurrence and development of the OC (**Supplementary Figure 2**).

### Construction of Prognostic Risk Model

**Figure 2:**
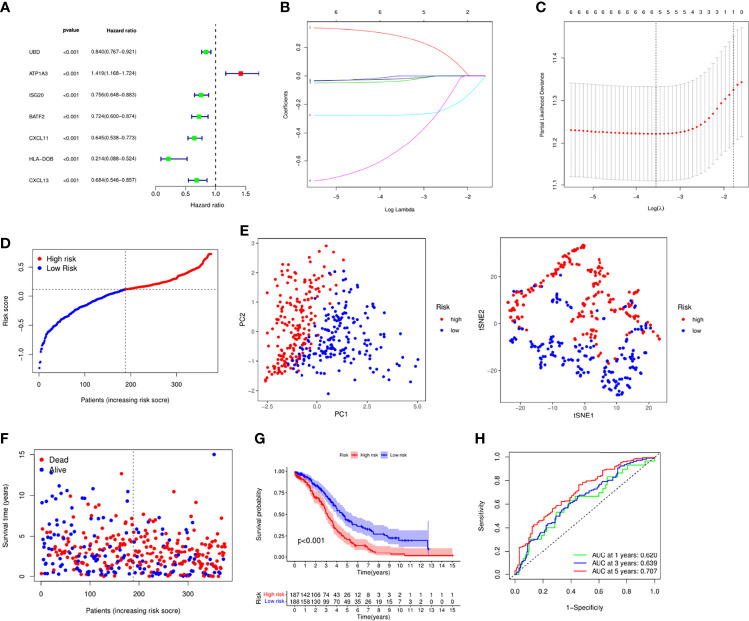
Establishment of risk signature in the TCGA cohort. **(A)** Univariate cox regression analysis of the necroptosis-related genes. **(B)** LASSO regression *via* the prognostic genes. **(C)** Cross-validation for tuning the parameter selection in the LASSO regression. **(D)** Distribution of the patients based the risk score. **(E)** PCA and tSNE analyses classified patients into two groups. **(F)** The survival status and risk score of each patient. **(G)** Survival analysis between high and low risk groups. **(H)** ROC analysis of the risk signature.

Next, we integrated gene expression profiles and patient survival information and constructed a risk model based on necroptosis-related genes. First, univariate Cox analysis was performed to screen out 7 prognostic-related necroptosis genes (*P* = 0.05). Among them, *UBD*, *ISG20*, *BATF2*, *CXCL11*, *HLA-DOB* and *CXCL13* were prognostic protective genes, while *ATP1A3* was a risk gene (**Figure 2A**). Then based on prognostic genes, we performed LASSO regression analysis to construct a 5-gene risk model, *UBD*, *ISG20*, CXCL*11*, *ATP1A3* and *HLA-DOB* (**Figures 2B, C**). The risk score formula is as follows: risk score = (−0.020* *UBD* exp.) + (0.278* *ATP1A3* exp.) + (-0.037* *ISG20* exp.) + (−0.275* *CXCL11* exp.) + (−0.517* *HLA-DOB* exp.). Based on the risk score, we divided 379 patients into high and low risk groups (**Figure 2D**). PCA and tSNE analysis results showed that patients could be well divided into high and low groups (**Figure 2E**). **Figures 2F**, **G** indicated that patients’ deaths in the high-risk group increased significantly, and the survival time was significantly shortened (*P* < 0.001). ROC analysis was used to verify the predictive performance of the model, and the results showed that the AUC was 0.620 at 1 year, 0.638 at 3 years, and 0.707 at 5 years, suggesting that the model had good sensitivity and specificity (**Figure 2H**).

### External Validation of the Model

To verify the robustness of this model, we included the GEO dataset. After normalizing the expression profiling data, we integrated the survival information of 360 OC patients and divided them into high and low risk groups based on the median risk score (**Supplementary Figure 3A**). Patients in high risk group had shorter survival time (**Supplementary Figures 3B, C**). KM survival analysis also confirmed that the low-risk group had better survival prognosis (**Supplementary Figure 3D**). The ROC analysis also proved the predictive power of the model (**Supplementary Figure 3E**).

### Independent Prognostic Analysis of the Risk Model

**Figure 3:**
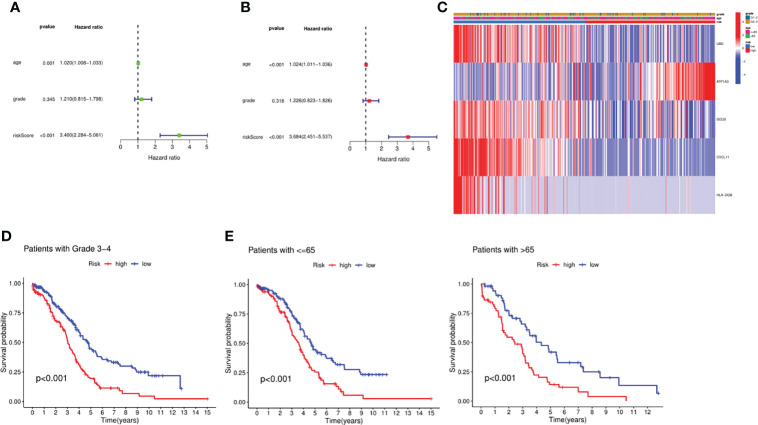
Independent prognosis analysis of the signature. **(A)** Univariate analysis for the TCGA cohort. **(B)** Multivariate analysis for the TCGA cohort. **(C)** Heatmap integrated by the expression profile and the clinical parameters. **(D)** Survival analysis between the groups with Stage III-IV. **(E)** Survival analysis between the groups with different age.

To confirm the independence of this risk model in clinical applications, we integrated patients’ clinical characteristics such as age and grade with risk scores, and calculated HR, CI and *P* for training and validation sets through univariate and multivariate COX analysis. In the training set, both univariate and multivariate COX analysis results indicated that age (HR=1.024, *P*<0.001) and risk score (HR=1.024, *P*<0.001) were independent risk factors as shown in **Figures 3A, B**. Not only that, we also integrated the expression levels of risk genes and presented them in the form of a heatmap (**Figure 3C**). According to the results of the heatmap, we found that in high-risk patients, the expressions of *UBD*, *ISG20*, *CXCL11*, and *HLA-DOB* were significantly decreased, while the expression of *ATP1A3* was increased in high-risk patients. Unfortunately, we found that age and grade did not appear to be significantly different between high and low risk. Nevertheless, we further performed a survival analysis on clinical characteristics, and we found that in patients younger than and older than 65 years and pathological grade 3-4, the survival prognosis of patients with low-risk scores was significantly better than that of high-risk groups. This also confirmed that the risk model we constructed was an independent risk factor (**Figures 3D, E**).

### Construction of the Nomogram and Model Comparison

**Figure 4:**
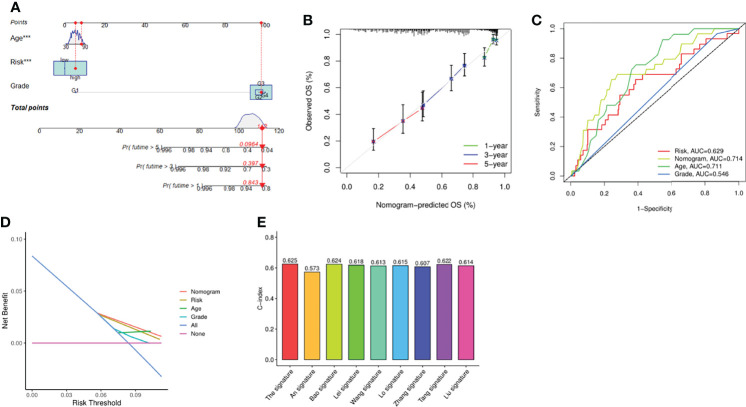
Evaluation of the risk signature. **(A)** Nomogram of the model integrated by the risk score and clinical parameters. **(B)** Calibration curve of the model. **(C)** ROC analysis of the nomogram and clinical features. **(D)** DCA curve based on the risk score and the clinical parameters. **(E)** Comparison between the signature and other established model. ***P <0.001.

To achieve the goal of establishing a clinical strategy for predicting the probability of survival in OC, the nomogram and calibration curves were generated based on the TCGA cohort to assess the probability of 1-, 3-, and 5-year OS (**Figures 4A, B**). The predictors of the nomogram consisted of 3 prognostic factors, including age, grade, and risk score. ROC analysis showed that the model had an AUC value of 0.629 for the risk score and 0.714 for the nomogram, which was higher than that of grade (AUC = 0.546) and patient age (AUC = 0.711) (**Figure 4C**). However, the AUC only measured the diagnostic accuracy of the predictive model and did not take the clinical utility of a particular model into account, so we also employed another method for evaluating clinical predictive models, diagnostic tests, and molecular markers, DCA. Based on the ggDCA package, we obtained DCA plots comparing age, class, risk score, and nomogram, and the results showed that the nomogram had better predictive power than risk score, age, and pathological grade (**Figure 4D**). Next, we also compared the accuracy and predictive ability of previously published risk models. By calculating and comparing the c index values ​​of various models, we found that the model was superior to the previously established models, which also demonstrated the promising predictive power of the model (**Figure 4E**).

### Difference Analysis Between High and Low Risk Groups

**Figure 5:**
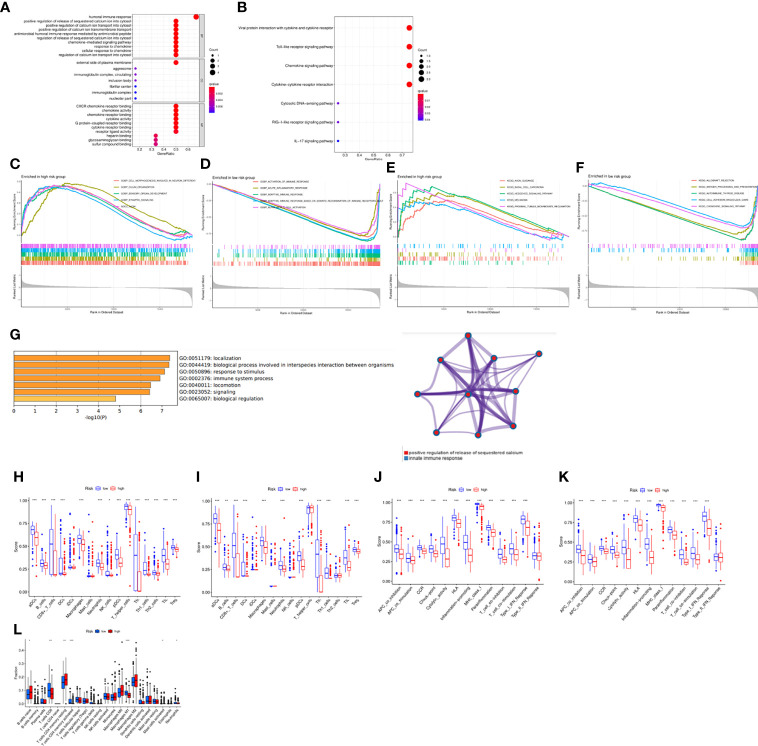
Functional analysis based on the DEGs between the two-risk groups in the TCGA cohort. **(A)** GO enrichment analysis. **(B)** KEGG pathways enrichment analysis. **(C–F)** GSEA analysis of the DEGs. **(G)** Functional analysis *via* “Metascape” website. **(H–K)** The abundance of the immune cells and functions between groups *via* ssGSEA. **(L)** Comparison of the enrichment of the immune cells *via* CIBERSORT. *P <0.05, **P <0.01 and ***P <0.001.

In order to further explore the gene expression and functional enrichment between the high and low risk groups, we firstly performed differential expression analysis on the high and low risk groups in the training set and validation set respectively as the criteria of log FC=1 and FDR=0.05, of which there were a total of 6 DEGs in the training set and 9 DEGs in the validation set. In-depth study of function enrichment analyses found that DEGs were enriched in chemokine receptor binding, chemokine activity function, chemokine signaling pathway, etc., which were closely related to the occurrence and development of OC (**Figure 5A**). Besides, it was also enriched in Toll-like receptor signaling pathway, RIG-I-like receptor signaling pathway and IL-17 signaling pathway, which were closely related to immunity, indicating that there might be significant differences in tumor immune microenvironment between high-risk and low-risk patients (**Figure 5B**). Except for that, GSEA enrichment analysis illustrated that in the high-risk group, cell morphogenesis involved in neuron differentiation, cilium organization, sensory organ development, synaptic signaling, and ason were significantly enriched (**Figure 5C**). While in the low-risk group, activation of immune response, acute inflammatory response, adaptive immune response, adaptive immune response based on somatic recombination of immune receptors and alpha beta T cell activation were significantly enriched (**Figure 5D**). Meanwhile, axon guidance, basal cell carcinoma, hedgehog signaling pathway, melanoma and proximal tubule bicarbonate reclamation were significantly enriched in the high- risk group (**Figure 5E**), while allograft rejection, antigen processing and presentation, hedgehog thyroid disease were significantly enriched in the high-risk group (**Figure 5F**). Eventually, metascape website was applied to explore the enriched gene function analysis, indicating that innate immune response and so on were noted (**Figure 5G**). Based on the above findings, we calculated the abundance of 13 types of immune cells and 16 types of immune functions enriched in each patient by ssGSEA, scored and compared the differences between high and low risk groups. It was found that among the 13 types of immune cells, except for iDCs and mast cells, all immune cells were significantly enriched in the low-risk group, and there were similar results in the immune function (**Figures 5H, I**). Except for type II IFN Response, the immune functions were all active in the low-risk group, and the same results were obtained in the validation set (**Figures 5J, K**). Furthermore, the abundance of infiltrating cells verified the above results *via* CIBERSORT (**Figure 5L**). Based on the immune infiltration in tumor, survival analyses were performed to investigate the role of immune cells and functions in patients’ prognosis (**Supplementary Figures 4**, **5**). These results indicated that the immune function in the tumor immune microenvironment of patients in the low-risk group functioned actively, with high expression of immune cells and robust anti-tumor ability, verifying the close relationship between the risk model and the tumor immune microenvironment.

### Associations Between the Model and Immune Cell Markers

To explore the different abundances of infiltrated immune cells between the two groups, the associations between the model and the gene markers. The risk model was significantly correlated with *CD8A*, *CD8B* for CD8^+^ T cell, *CD4* for CD4^+^ T cell, *CD80*, *CD86* for M1 macrophage, *CD163* for M2 macrophage, *CD14*, *CD33* for Monocyte, *CD19*, *CD79A* for B cell, *HLA-DRA* for Dendritic cell, *BCL6* for Tfh, *STAT1*, *STAT4* for Th1, *FOXP3*, *STAT5B* for Tregs. It was suggested by these findings that the risk model was strongly correlated with tumor immune microenvironment since it was linked with immune cells (**Supplementary Figures 6A, B**).

### Deciphering Mutational Spectrum

**Figure 6:**
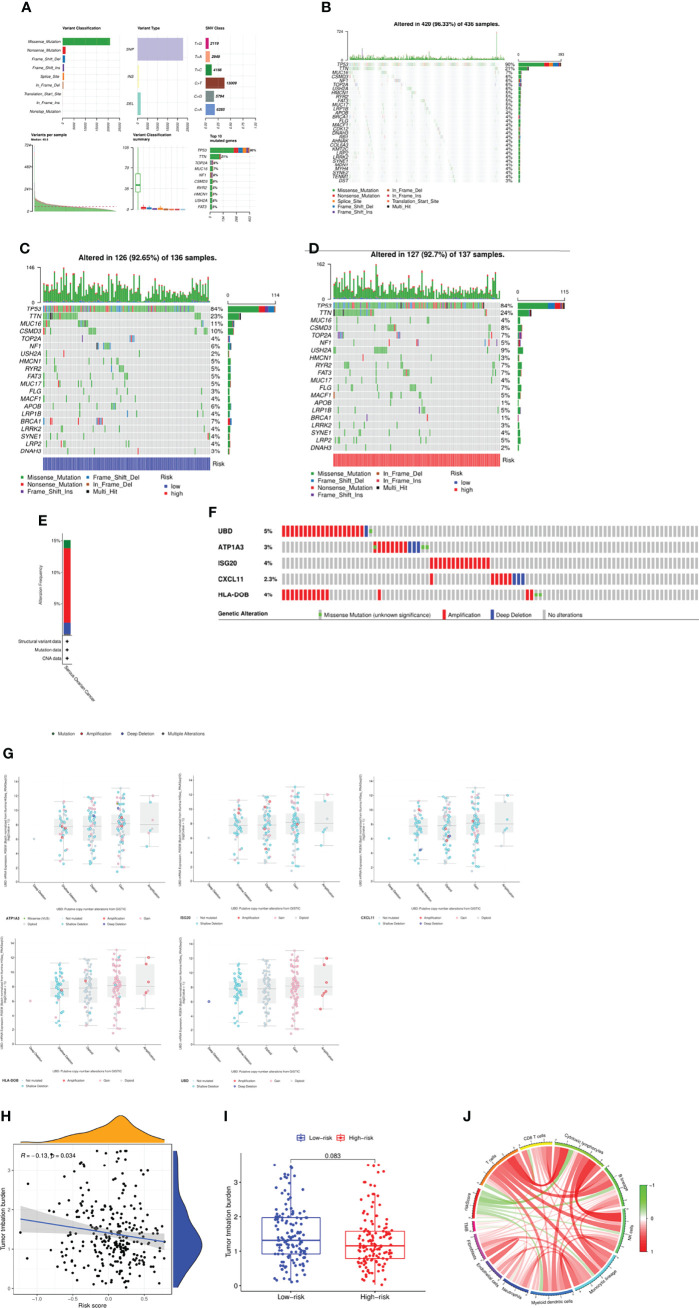
Mutation spectrum of the patients between two groups. **(A, B)** Mutation profile of the patients in TCGA cohort. **(C)** Mutation profile of the patients in low-risk group. **(D)** Mutation profile of the patients in high-risk group. **(E)** Frequency of mutation in hub genes in serous ovarian cancer. **(F, G)** Mutation of each hub gene. **(H)** Correlation between TMB and risk score. **(I)** Comparison of TMB between two groups. **(J)** Link between the TMB, risk score and the immune cells.

We next studied the mutation spectrum of all patients. Among the 436 samples, 420 samples had gene mutations (96.33%). The most common mutation classification was missense mutation, and the most common mutation type was SNP, followed by DEL, and finally INS. Besides the most SNV class is C>T mutation (**Figure 6A**). The top three genes with the highest mutation probability were *TP53*, *TTN* and *MUC16* (**Figure 6B**). Further, we also compared the mutation status of patients in the high and low risk groups. There was a certain difference between the two groups. In the low risk group, the top three genes with mutation probability were *TP53* (84%), *TTN* (23%) and *MUC16* (11%) (**Figure 6C**), while the top three genes with mutation probability in the high-risk group were *TP53* (84%), *TTN* (24%) and *USH2A* (9%) (**Figure 6D**). In addition, we searched the mutation status of 5 risk genes on the cBioPortal website. The gene mutation frequencies from high to low are *UBD*, *ISG20*, *HLA-DOB*, *ATP1A3* and *CXCL11* (**Figure 6E**). Among them, amplification was the most common type of mutation, followed by missense mutation, and finally deep deletion (**Figures 6F, G**). Tumor mutational burden (TMB) was defined as the total number of somatic gene coding errors, base substitutions, and gene insertion or deletion errors detected per mega base. Therefore, we calculated the TMB of all samples and analyzed the relationship between TMB and risk score, and found that TMB was significantly negatively correlated with risk score (R=-0.13, *P*=0.034) (**Figure 6H**), but there was no obvious difference between the two groups (*P*=0.083) (**Figure 6I**). Eventually, we deeply investigated the link between the TMB, risk score and the immune cells (**Figure 6J**). Not surprisingly, there was significant correlation between them, suggesting that the risk model might have a promising prospect for immunotherapy.

### CNV of the Necroptosis-Related Genes in OC

Genomic alterations were characteristic of cancer, and different kinds of cancers were characterized as specific aberrations that provided clues to the cause and prognosis of disease. Abnormal DNA copy number changes (CNVs) were an important molecular mechanism in many human diseases (cancer, hereditary diseases, cardiovascular diseases). Therefore, we explored the CNV landscape of the necroptosis-related genes in OC *via* UCSC (https://xena.ucsc.edu/), which demonstrated that nearly all genes had CNV referred to the loss or gain of copies of a genomic DNA region in OC. The most frequent gain of function of gene was *MYC*, while the loss was *SIRT3* (**Supplementary Figure 7A**). Not only that, we also depicted the spectrum of these genes *via* RCircos presented a circos plot (**Supplementary Figure 7B**).

### Tumor Immune Landscape and Tumor Immunogenicity

**Figure 7:**
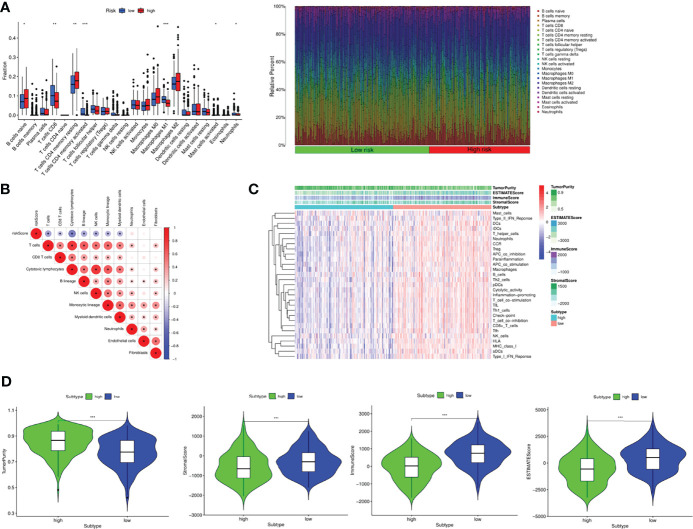
Tumor immune landscape in OC. **(A)** Bar plot of 24 kinds of immune cells between high and low risk groups *via* CIBERSORT. **(B)** The correlation between immune cells and risk score. **(C)** Heatmap integrated by the immune scores and immune cells and functions. **(D)** Comparison of tumor purity, stromal score, immune score and ESTIMATE scores between the two groups. *P <0.05, **P <0.01 and ***P <0.001.

CIBERSORT was used to assess immune cell infiltration in different risk groups and the results were visualized by bar plot. Naive B cells, CD8^+^T cells, T cells CD4 memory resting, T cells CD4 memory activated, M1 Macrophages, Mast cells activated and Neutrophils were enriched in different risk groups. The levels of memory CD8^+^T cells, M1 Macrophages and Neutrophils were higher in the low-risk group than in the high-risk group. The levels of Naive B cells, T cells CD4 memory resting, T cells CD4 memory activated and Mast cells activated were higher in the high-risk group than in the low-risk group (**Figure 7A**). The correlation analysis plot showed that immune cells such as T cells, CD8^+^T cells, Cytotoxic lymphocytes, B cells, NK cells, Monocytic cells and Myeloid dendritic cells were significantly negatively correlated with the risk score (**Figure 7B**). Next, survival analysis was performed to explore the relationship between high and low levels of immune cells and patient prognosis. The results showed that high levels of M1 macrophages (*P*<0.001), T cells CD4 memory activated (*P*<0.001), T cells follicular helper (*P*<0.001), T cells gamma delta (*P*=0.002) had significantly higher prognosis than low-level, low-level M2 macrophages (*P*=0.028), Mast cells activated (*P*=0.038), Monocytes (*P*=0.002), Neutrophils (*P*=0.006), Plasma cells (*P*=0.011), T cells CD4 memory resting (*P*=0.028), Tregs (*P*=0.028) had significantly better prognosis than highly enriched patients (**Supplementary Figure 8**). Meanwhile, we also studied the relationship between immune function enrichment and survival prognosis, and found that high levels of aDCs (*P*=0.004), APC co inhibition (*P*<0.001), B cells (*P*=0.005), CCR (*P*=0.006) and other immune cells (**Supplementary Figure 9**). The functional prognosis was significantly higher than the low level, which were also consistent with those of the ssGSEA analysis. We next calculated the immune score, mesenchymal score, tumor purity, and ESTIMATE score for each patient using the estimate package, and compared the differences between patients in the high- and low-risk groups (**Figure 7C**). The results showed that the tumor purity of patients in the high-risk group was significantly higher than that of the low-risk group, while the stromal score, immune score, and ESTIMATE score were significantly higher in the low-risk group than the high-risk group, suggesting that the patients in the low-risk group had active immunity and strong anti-tumor immunity with a better prognosis (**Figure 7D**).

### Evaluation of the Response to Immunotherapy

**Figure 8:**
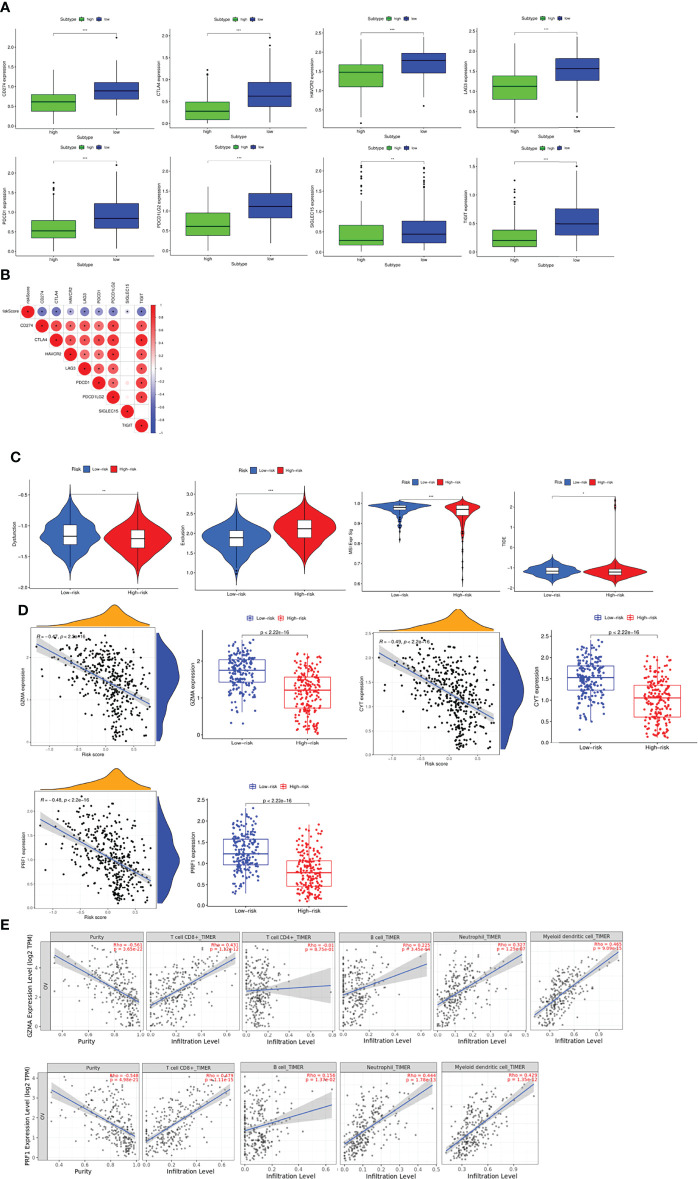
Evaluation of the sensitivity to immunotherapy **(A)** Expression profiles of immune checkpoint molecules between groups. **(B)** Correlation between risk score and immune checkpoint molecules. **(C)** Evaluation of the sensitivity to immunotherapy *via* TIDE score. **(D)** Evaluation of the immunogenicity and the favorable immune TME of patients *via* CYT score. **(E)** Pearson analyses between the expression of GZMA, PRF1 and tumor purity, immune cells *via* TIMER. *P <0.05, **P <0.01 and ***P <0.001.

Based on the above results, it was found that patients in the different risk groups had different immune landscape, indicating that patients responded to the immunotherapy in different degrees. Therefore, we investigated the relationship between risk scores and expression of common immune checkpoints in OC. We found that the expressions of *CD274*, *CTLA4*, *HAVCR2*, *LAG3*, *PDCD1*, *PDCD1LG2*, *TIGIT* and *SIGLEC15* were significantly increased in the low-risk group (**Figure 8A**), and the correlation analysis further proved that the expression of these immune checkpoint molecules was negatively correlated with the risk score (**Figure 8B**). Finally, we used the TIDE algorithm to explore the response of patients in the high- and low-risk groups to immunotherapy. The results found that the Dysfunction, MSI and TIDE scores in the low-risk group were significantly higher than those in the high-risk group, and the Exclusion score was significantly lower than that in the high-risk group, suggesting that patients in the low risk group were less effective with immune checkpoint inhibition, while those in the high-risk group benefited more (**Figure 8C**). Subsequently, immune cytolytic activity (CYT) based on the expression levels of granzyme A (GZMA) and perforin 1 (PRF1), which were significantly upregulated with cytotoxic T cell activation, was utilized to estimate the immunogenicity and the favorable immune TME of patients. The results showed that the expression of *GZMA*, *PRF1* and CYT score were significantly increased in low risk group and negatively correlated with risk score, suggesting enhanced immune activity and better OS (**Figure 8D**). Furthermore, the relationship between the expression of *GZMA*, *PRF1* and tumor purity and immune cells was investigated, which indicated the similar findings (**Figure 8E**).

### Chemosensitivity

Chemotherapy drugs were very indispensable for the treatment of OC, and the main indicator that affected the efficacy of drug treatment was drug sensitivity. Therefore, we analyzed the drug sensitivity of patients in the high and low risk groups to common chemotherapeutic drugs. The IC50 of Imatinib and AG.014699 were significantly higher than that of the high-risk group, and the sensitivity was worse than that of the high-risk group. The IC50 of ATRA, Bortezomib, Cyclopamine, Dasatinib, Metformin, Methotrexate, Paclitaxel, Parthenolide were significantly lower than that of the high-risk group, and the sensitivity was better than that of the high-risk group (**Supplementary Figure 10A**). We then investigated the correlation between core genes and chemotherapeutic drug sensitivity, which indicated that the expression of *HLA-DOB* was positively correlated with the sensitivity of Nelarabine, Dexamethasone, Fluphenazine, Cyclophosphamide, Arsenic trioxide, Fludarabine, Vorinostat and Axitinib, the expression of *ATP1A3* was positively correlated with Nelarabine, Fludarabine and Digoxin, and the expression of *ISG20* was positively correlated with Dabrafenib, Vemurafenib, and Denileukin Diftitox Ontak, negatively correlated with Dasatinib (**Supplementary Figure 10B**). Subsequently, we identified the potential drugs *via* the DEGs between high and low risk groups, and found that several drugs targeted *CXCL10* such as NI-0801, ELDELUMAB and so on. Not only that, we searched and obtained the 2D and 3D structure of the targeted drugs (**Supplementary Figure 11**). Eventually, cancer stem cells referred to cells in tumors that had self-renewal ability and could generate heterogeneous tumor cells, which were closely related to tumor survival, proliferation, metastasis and recurrence. Stemness index was an indicator of the similarity between tumor cells and stem cells. Therefore, we ought to figure out the relationship between risk score and stemness. RNAss and DNAss were indices calculated based on RNA expression data and methylation data, respectively, which were between 0 and 1. The larger the value, the lower the degree of cell differentiation and the stronger the stem cell characteristics. However, there was no difference between them (**Supplementary Figure 10C**).

### Evaluation of Immunotherapy

**Figure 9:**
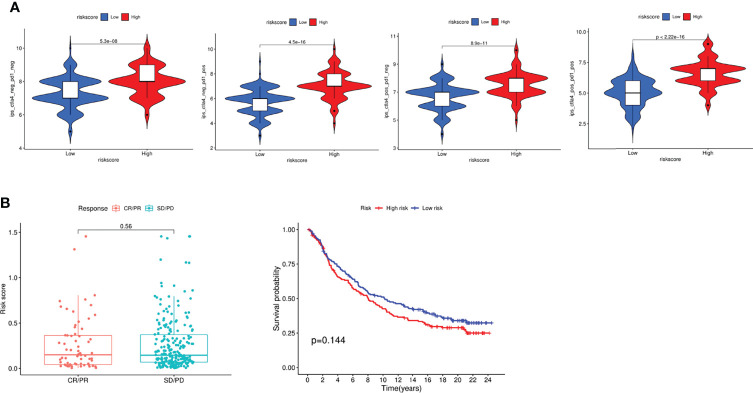
Evaluation of Immunotherapy to CTLA-4 and PD-1. **(A)** Evaluation of the IPS score in different groups. **(B)** Response to immunotherapy and survival analysis in different groups.

Based on the above results, we next performed analyses to estimate the responsiveness of CTLA-4 and PD-1 immunotherapy *via* the public dataset TCIA (The Cancer Immunome Atlas, https://tcia.at/home). Interestingly, the findings showed that patients in high risk group responded more sensitively to the low risk group *via* the CTLA-4 and PD-1 immunotherapy (**Figure 9A**). Finally, we investigated the association of immunotherapy with risk scores using previous datasets, such as IMvigor, but unfortunately, we found no significant difference in risk scores between immunotherapy-responsive and ineffective groups (**Figure 9B**).

### Expression Levels of Key Genes in Cells and Clinical Samples

**Figure 10:**
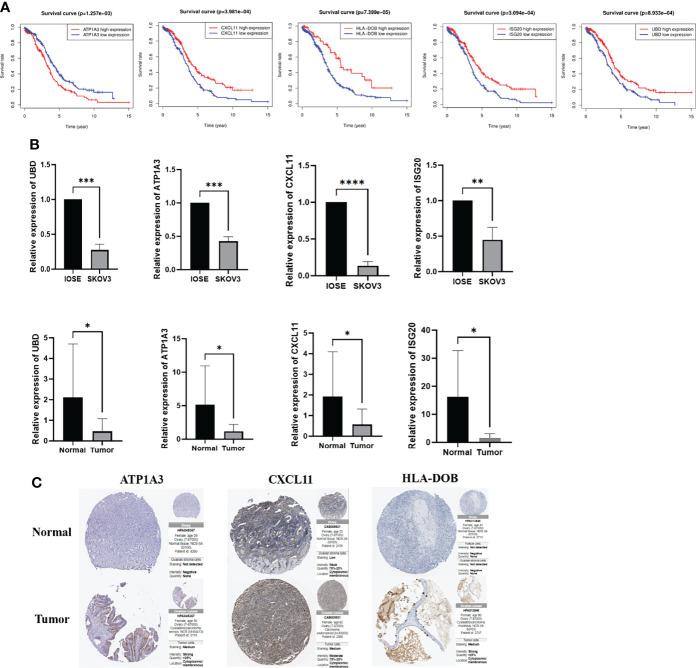
Survival analyses and the mRNA expression level of the key genes in the clinical samples and cells. **(A)** Kaplan–Meier curves for comparison of the OS between low- and high-expression groups **(B)** Comparison of the mRNA expression of the key genes in the clinical samples and cells **(C)** Comparison of the protein expression of the key genes in the Human Protein Atlas. *P < 0.05, **P < 0.01, ***P < 0.001 and ****P < 0.0001.

First, we performed analysis to identify the role of the key genes in OC, and the results indicated that high expression of *CXCL11*, *HLA-DOB*, *ISG20* and *UBD* predicted better prognosis, while low expression of *ATP1A3* indicated worse prognosis (**Figure 10A**). To verify the expression level of the key genes, qRT-PCR was performed to explore the clinical significance between IOSE and SKOV3 cells, normal and tumor tissues. The results indicated that the mRNA expression levels of *UBD*, *ATP1A3*, *ISG20* and *CXCL11* are lower in SKOV3 and tumor samples than IOSE and normal tissues, which was consistent with the results of TCGA cohort, while *HLA-DOB* showed no difference, indicating the indispensable role of the key genes in the occurrence and development of OC (*: *P*<0.05, **: *P*<0.01, ***: P*<*0.001, ***: *P*<0.0001) (**Figure 10B**). Eventually, we also explored the protein expression of the candidate genes in Human Protein Atlas, and the results was similar with the previous results, while *UBD* and *ISG20* were not retrieved (**Figure 10C**).

## Discussion

Evidences suggested that necroptosis played a crucial role in tumorigenesis, tumor progression, and tumor immune regulation (4). So far, the net effect of necroptosis in cancer had not been determined, and the tumor effect of promoting or anti-necroptosis might depended on the cell type and stage of the cancer (14). On the one hand, necroptosis could promote tumorigenesis and progression by triggering the tumor-promoting tumor immune microenvironment and regulating stromal cell responses (15–17). However, in some cases, necroptosis could also inhibit tumor progression and experimental studies on tumor immunology have found that necroptosis might play a central role in triggering immunogenicity and promoting natural or therapeutically driven anticancer immune surveillance (18).

A number of previous studies have shown that necroptosis plays an important role in various tumors, and inhibition of necroptosis in breast cancer significantly promoted malignant biological behaviors such as cell proliferation and migration (19). In gastric cancer, induction of necroptosis inhibited the migration and invasion of gastric cancer (20). Not only that, Wang et al. performed a comprehensive bioinformatics analysis in gastric cancer, and constructed a prognostic model of necroptosis, and identified the lncRNA SNHG1/miR-21-5p/TLR4 regulatory axis to demonstrate the role of necroptosis (21).

In our study, firstly, we identified the expression of necroptosis genes in OC, and found that most necroptosis-related genes were differentially expressed, suggesting that necroptosis was involved in the occurrence and development of OC. Stepwise GO and KEGG analysis based on these DEGs indicated that these key genes were mainly involved in tumor necrosis factor-mediated signaling pathway, NF-KB signaling pathway. Accumulating evidence revealed that NF-kappa B signaling pathway played a vital role in inflammation and cancer progression (22). Moreover, TNF signaling pathway was also found to be involved in balancing cell survival and necroptosis (23).

Then we constructed a 5-gene risk model, *UBD*, *ISG20*, *CXCL11*, ATP1A3 and *HLA-DOB*, which could well predict the survival prognosis of patients. Independent analysis also clearly proved that the model was an independent risk factor for OC patients.

*UBD* is a ubiquitin-like protein, and its function of targeting protein degradation is similar to that of ubiquitin, which is also the only known ubiquitin-like protein that can directly mediate ubiquitin-independent proteasomal degradation (24). Studies have shown that *UBD* is overexpressed in CRC tumor tissue, and its overexpression is positively correlated with tumor size and TNM stage in CRC patients. Functionally, *UBD* significantly accelerated CRC cell viability and proliferation *in vitro* and promoted tumorigenesis *in vivo* (25). Increased *UBD* expression was also found in breast cancer tissues. Overexpression of *UBD* was explored to be associated with epirubicin resistance in TNBC *in vitro*. Furthermore, *UBD* was highly expressed in TNBC compared to non-TNBC, which played a positive role in epirubicin resistance, suggesting a poor prognosis for TNBC treatment. But the role of *UBD* in OC was currently unclear. In this study, *UBD* is a prognostic favorable gene of OC, which is contrary to previous studies. Therefore, in-depth mechanism studies are still needed to explore the molecular pathways of OC.

Located on human chromosome 15q26, *ISG20* is an RNA exonuclease that cleaves single-stranded RNA and DNA (26). Previous studies have found that *ISG20* plays a role in mediating the antiviral activity of interferon and controlling cell proliferation and differentiation (27, 28). A study in clear cell renal cell carcinoma identified *ISG20* as a potential biomarker and therapeutic target for clear cell renal cell carcinoma which promoted cell proliferation and metastasis (29). Not only that, *ISG20* was also screened in the OC glycolysis gene model constructed by Yu et al. It is differentially expressed in ovarian cancer and was associated with patient survival prognosis, but its specific molecular mechanism as a necroptosis gene had not been elucidated (30).

Chemokine ligand CXC motif chemokine ligand 11 (*CXCL11*), also known as IFN-inducible T cell alpha chemokine, primarily mediates the recruitment of T cells, natural killer (NK) cells, monocytes and macrophages at the site of infection *via* cognate G protein-coupled receptors CXCR3, such as CXCL9 and CXCL10 (31, 32). This signaling axis has been implicated in a variety of physiological activities, including immune cell migration, differentiation, and activation. *CXCL11* is involved in the progression of various cancers. Upregulation of *CXCL11* was associated with better prognosis in colon adenocarcinoma, which promoted antitumor immunity to benefit survival, identified as an independent prognostic biomarker in colon adenocarcinoma patients. In OC, downregulation of *CXCL11* restrained angiogenesis and tumor growth, however, in our study, *CXCL11* predicted better prognosis, contrary to previous studies, so further studies were still needed to explore the mechanism.

Na+/K+ ATPase is a heterotrimeric α-β-γ protein complex, in which four alpha isoforms (α1-4) express in humans, encoded by the ATP1A 1-4 genes. The α3 isoform encoded by *ATP1A3* located on chromosome 19q, which expressed almost exclusively in neurons (33). At present, there is no research on the relationship between *ATP1A3* and tumor, and our research shows that *ATP1A3* may be involved in tumorigenesis and development as an oncogene of ovarian cancer, so above results may be a new direction for future research.

In terms of *HLA-DOB*, it belongs to the HLA class II beta chain paralogues. This class II molecule is a heterodimer consisting of an alpha (DOA) and a beta chain (DOB), both anchored in the membrane. At present, there are few studies on the involvement of *HLA-DOB* in tumor mechanisms. The expression of *HLA-DOB* in multiple myeloma was significantly higher than that in normal plasma cells, suggesting that it was a potential target for immunotherapy (34). Meanwhile, a bioinformatic analysis of high-grade serous OC by Chang et al. showed that *HLA-DOB* might act as a prognostic protective gene and participate in the composition of the risk model, which was consistent with our study (35).

Over the past few years, the link between necroptosis and tumors has been gradually elucidated, but the underlying regulatory role between tumor immunity and necroptosis remains elusive. Therefore, we analyzed the differences between high- and low-risk groups to find out the specific mechanism. Functional analysis suggested that DEGs were enriched in chemokine receptor binding, chemokine activity function, chemokine signaling pathway, etc., which was closely related to the occurrence and development of OC. Not only that, the immune pathways are significantly enriched in the low-risk group, suggesting that there are significant differences in the tumor immune microenvironment between patients in the high- and low-risk groups, further indicating that the model is closely associated with immunity. On this basis, we identified the expression levels of immune cells and immune functions in the high- and low-risk groups, and the results were consistent with the previous ones, clearly indicating that patients in the low-risk group had high immune levels and strong anti-tumor immunity, indicating better prognosis.

Notably, the complex interaction between tumor cells and the tumor microenvironment not only plays a pivotal role in tumor development, but also has a significant impact on patient immunotherapy efficacy and overall survival. It has been reported that the intratumoral and peritumoral distribution of immune cells, the composition of immune cells, and the overall immune environment and histology of breast tumors can affect not only the degree of tumor malignancy, but also the effect of immunotherapy (36, 37). The high immune infiltration in the low-risk group partly reflects the lower degree of malignancy and better response of various treatments, which means that our signature can not only differentiate the survival prognosis of patients, but also reflect the level of immune infiltration cells.

Assessing the tumor immune microenvironment is helpful to understand the molecular characteristics of patients with different types of tumors, and more importantly, it may provide more individualized treatments for different patients. Therefore, we next identified the expression of immune checkpoint molecules and potential responses to immunotherapy in patients with high and low risk groups. Immune checkpoint molecules are inhibitory pathways in the immune system that are regulated by ligand/receptor interactions, which plays an important role in maintaining autoimmune tolerance and regulating the duration and magnitude of physiological immune responses, thereby avoiding damage and destruction of normal tissues by the immune system. The high expression of immune checkpoint molecules in tumor cells is closely related to tumor immune escape. Our results suggested that the expression of immune checkpoint molecules is significantly increased in the low-risk group, which may demonstrate that patients in the low-risk group have a high possibility of immune escape. Currently, one of the most promising approaches to activate therapeutic antitumor immunity is to block immune checkpoints. The TIDE score is an algorithm used to assess the ability of a potential immune checkpoint to block the immune response, and the TIDE score serves as a valid surrogate for traditional single biomarkers for predicting ICB response. A higher TIDE score not only indicates that the tumor has an immune escape phenotype, but also predicts a poorer response to ICB in cancer patients (38). Similar to the previous results, our results found that patients in the high-risk group were significantly more responsive to immunotherapy than those in the low-risk group, indicating that ICB therapy in the high-risk group could effectively enhance the original antitumor immune activity and provide a durable immune response.

At present, immunotherapy has become a new treatment strategy for OC, but in clinical practice, the effect of immunotherapy on OC is still limited. Therefore, our above results provide a certain possibility for OC immunotherapy. Notably, combination therapy is playing an increasingly important role because single immunotherapy is not ideal for patients. In this manuscript, the sensitivity of Metformin, Methotrexate, Paclitaxel and other drugs for patients in the low-risk group was better than that in the high-risk group, suggesting that the patients in the low-risk group might benefit from chemotherapy. Therefore, the constructed model could better analyze the sensitivity to chemotherapy and immunotherapy in high and low risk groups, which also provided the possibility of combining the two treatment strategies in OC, such as the combination of immunotherapy with chemotherapy or PARP inhibitor, etc.

However, this study has several limitations. First, our prognostic model was constructed and validated using retrospective data from public databases. More prospective real-world data are needed to validate its clinical utility. Second, the link between the model and other clinical characteristics was not assessed, and third, the association between risk score and immune activity has not been experimentally resolved. Finally, because there are no enough gene expression data from patients receiving immunotherapy, prospective studies are needed to validate the ability of the model to predict immunotherapy response.

In conclusion, we constructed a 5-gene model based on necroptosis-related genes, which could not only effectively predict the survival and prognosis of OC patients as an independent risk factor, but also reveal a close correlation with the tumor microenvironment and immunotherapy, providing sensitive and effective biomarkers and a new direction for personalized immunotherapy.

## Data Availability Statement

The data sets used in this study can be downloaded from GTEX, TCGA and GEO and the names of the repository/repositories and accession number(s) can be found in the article.

## Ethics Statement

This study was approved by the ethics committee of the Renmin Hospital of Wuhan University (WDRY2020-K218).

## Author Contributions

YC and WH designed the study. ZW, GC and FD collected the clinical information, gene expression data, and data analysis. ZW and GC wrote the manuscript and SL provided revisions. All authors contributed to the article and approved the submitted version.

## Funding

This study was supported by the China Medical Association Clinical Medical Research Special Fund Project (grant number 17020310700); National Natural Science Foundation of China (grant number 82071655, 81860276); Key Research and Development Program of Hubei Province (2020BCB023); the Fundamental Research Funds for the Central Universities (grant number 2042020kf1013); Educational and Teaching Reform Research Project (grant number 413200095); Graduate credit course projects (grant number 413000206).

## Conflict of Interest

The authors declare that the research was conducted in the absence of any commercial or financial relationships that could be construed as a potential conflict of interest.

## Publisher’s Note

All claims expressed in this article are solely those of the authors and do not necessarily represent those of their affiliated organizations, or those of the publisher, the editors and the reviewers. Any product that may be evaluated in this article, or claim that may be made by its manufacturer, is not guaranteed or endorsed by the publisher.

## Supplementary Material

Supplementary Figure 1 Identification of the differentially expressed necroptosis-related genes **(A)** Heatmap (green: low expression; red: high expression) of the necroptosis-related genes between the normal and the tumor tissues. **(B)** PPI network illustrated the interactions of the differentially expressed necroptosis-related genes **(C)** The correlation network of the necroptosis-related genes (red line: positive correlation; blue line: negative correlation **(D)** KEGG pathways enrichment analysis **(E)** GO function enrichment analysis.

Supplementary Figure 2 The identified key genes via random Forest.

Supplementary Figure 3 Validation of the risk signature in the GEO cohort **(A)** Distribution of the patients based the median risk score in the TCGA cohort **(B)** PCA and tSNE analyses classified patients into two groups in GEO cohort **(C)** The risk score and survival status of each patient in GEO cohort **(D)** Kaplan–Meier curves for comparison of the OS between low- and high-risk groups **(E)** The AUC values of the risk model in TCGA.

Supplementary Figure 4 Survival analysis of different abundance of the immune cells.

Supplementary Figure 5 Survival analysis of different abundance of the immune functions.

Supplementary Figure 6 Association between the model and immune cells **(A)** The expression profile of the immune cell markers and the correlation with the model between high and low risk groups **(B)** The association between the risk score and immune cell markers.

Supplementary Figure 7 CNV of the necroptosis-related genes in OC**(A)** CNV frequency of the necroptosis-related genes **(B)** Circos plot of the CNV spectrum.

Supplementary Figure 8 Survival analysis between high and low degrees of immune cells.

Supplementary Figure 9 Survival analysis between high and low degrees of immune functions.

Supplementary Figure 10 Chemosensitivity and stemness of different groups **(A)** IC50 of multiple chemotherapy drugs between groups**(B)** Correlation between the risk genes and chemotherapy drugs **(C)** Assessment of the association of stemness and risk score.

Supplementary Figure 11 The structure of predicted targeted drugs based on the DEGs in different groups.

## Abbreviations

OC, ovarian cancer; GTEX, The Genotype-Tissue Expression; TCGA, The Cancer Genome Atlas; GEO, Gene Expression Omnibus; FDR, false discovery rate; DEGs, differentially expressed genes; OS, overall survival; AUC, area under curve; CNV, Copy-number variant; PFS, progression free survival; KEGG, Kyoto Encyclopedia of Genes and Genomes; GO, Gene Ontology; KM, Kaplan-Meier.
