# Supplementary figures and images for: Identification and Verification of Necroptosis-Related Gene Signature With Prognosis and Tumor Immune Microenvironment in Ovarian Cancer
Source: Front Immunol. 2022 Jun 24;13:894718. doi: 10.3389/fimmu.2022.894718 (PMC9265217; doi:10.3389/fimmu.2022.894718)

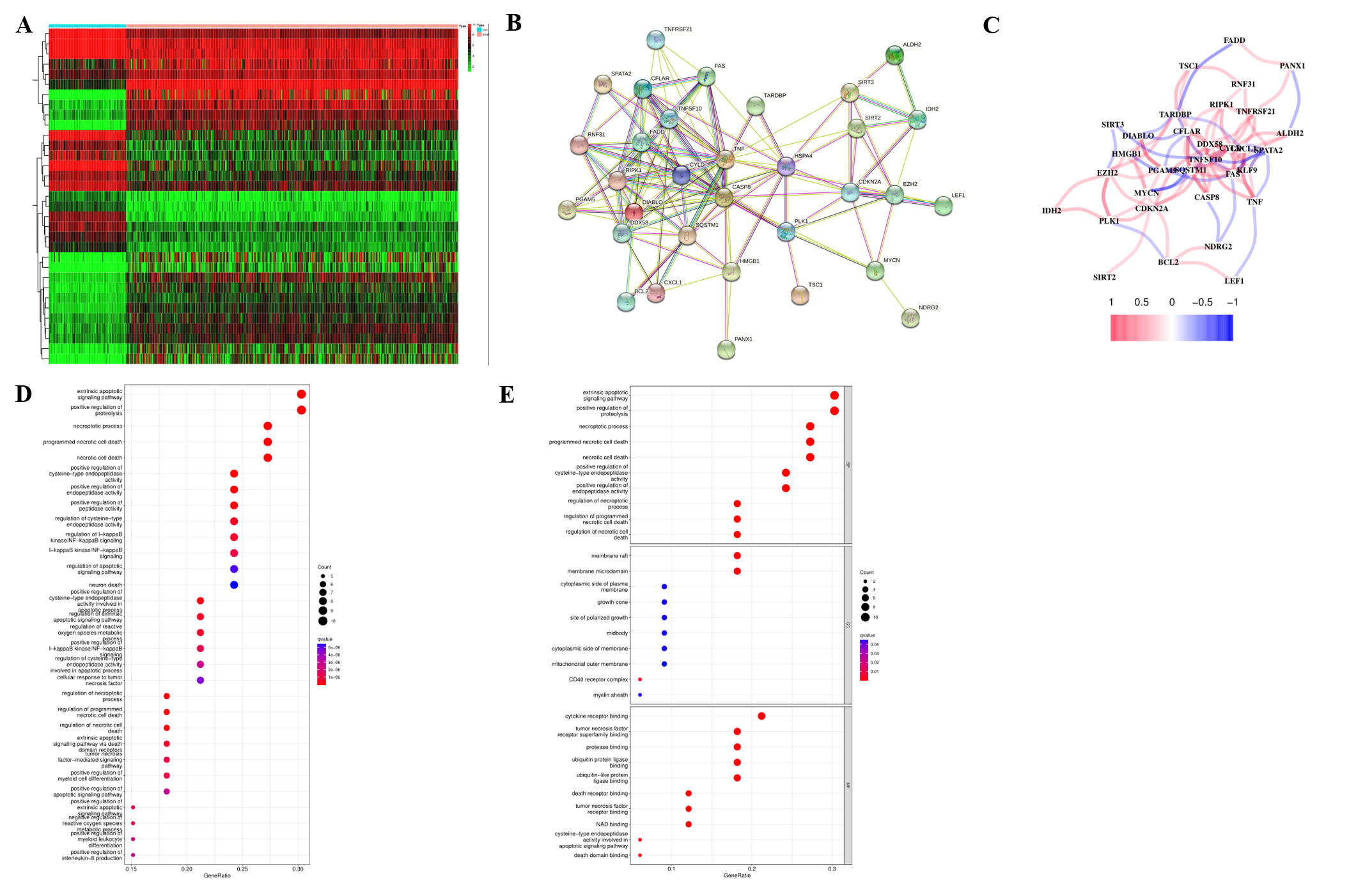

Supplement: Supplementary Figure 1 — Identification of the differentially expressed necroptosis-related genes (A) Heatmap (green: low expression; red: high expression) of the necroptosis-related genes between the normal and the tumor tissues. (B) PPI network illustrated the interactions of the differentially expressed necroptosis-related genes (C) The correlation network of the necroptosis-related genes (red line: positive correlation; blue line: negative correlation (D) KEGG pathways enrichment analysis (E) GO function enrichment analysis. [file Image_1.tif]

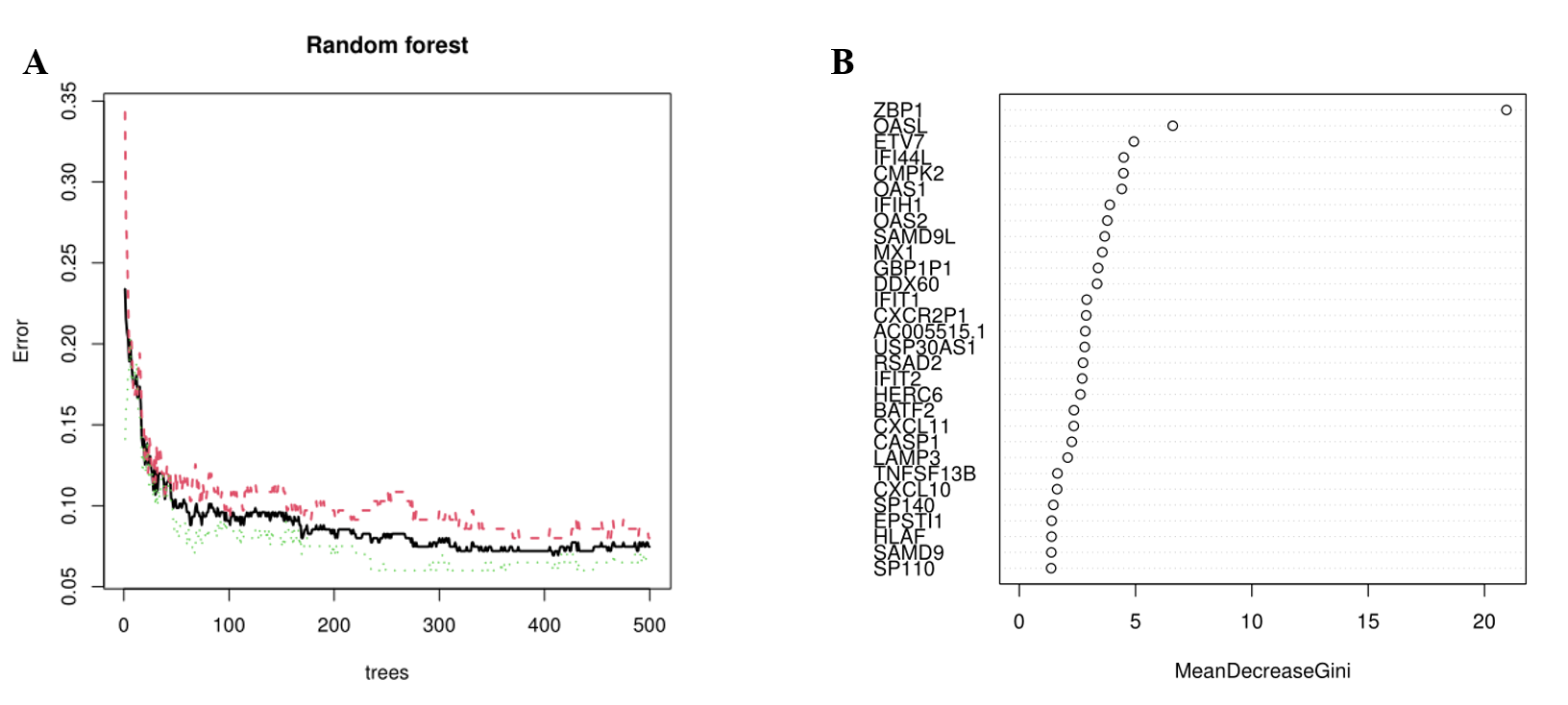

Supplement: Supplementary Figure 2 — The identified key genes via random Forest. [file Image_2.tif]

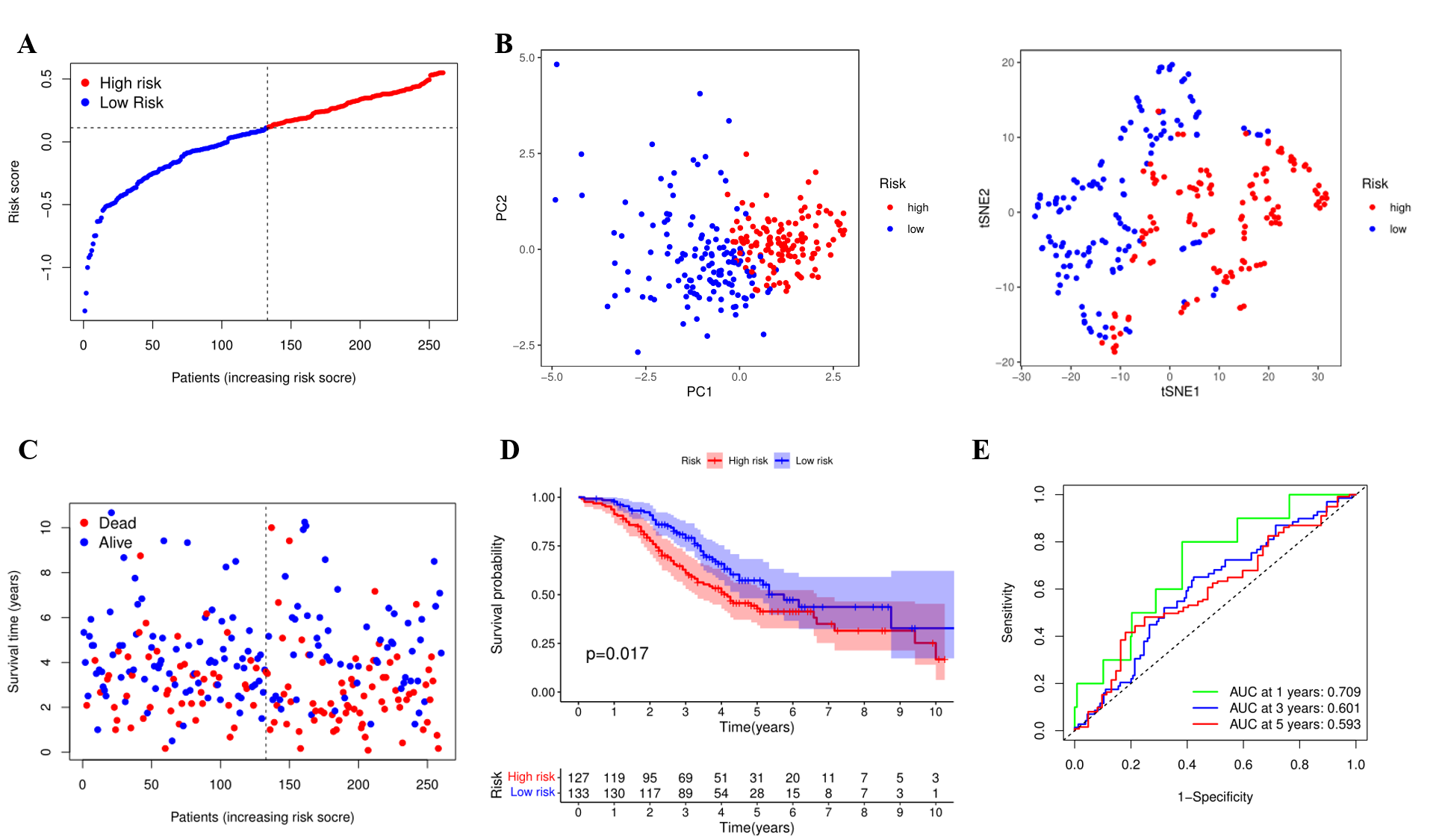

Supplement: Supplementary Figure 3 — Validation of the risk signature in the GEO cohort (A) Distribution of the patients based the median risk score in the TCGA cohort (B) PCA and tSNE analyses classified patients into two groups in GEO cohort (C) The risk score and survival status of each patient in GEO cohort (D) Kaplan–Meier curves for comparison of the OS between low- and high-risk groups (E) The AUC values of the risk model in TCGA. [file Image_3.tif]

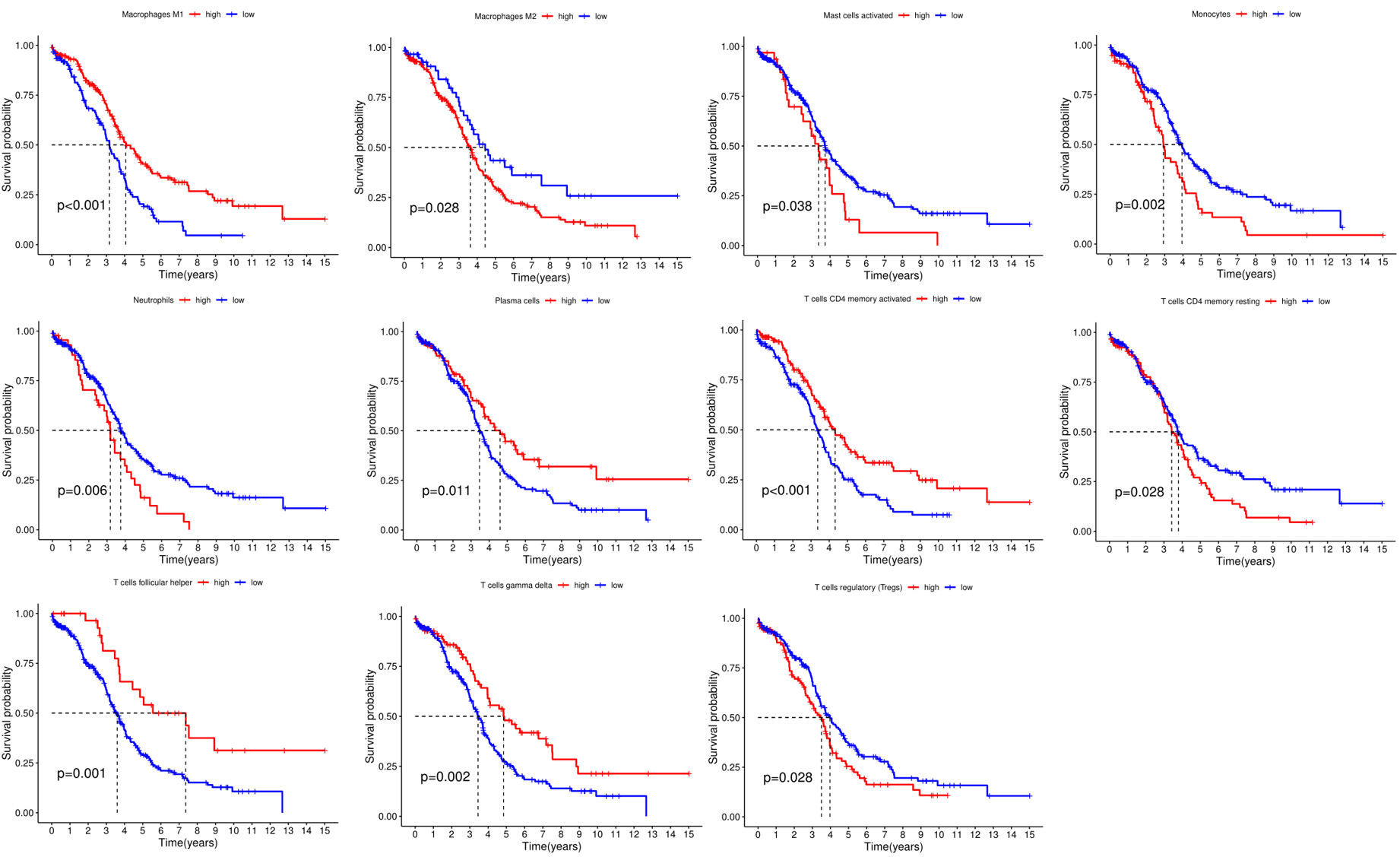

Supplement: Supplementary Figure 4 — Survival analysis of different abundance of the immune cells. [file Image_4.tif]

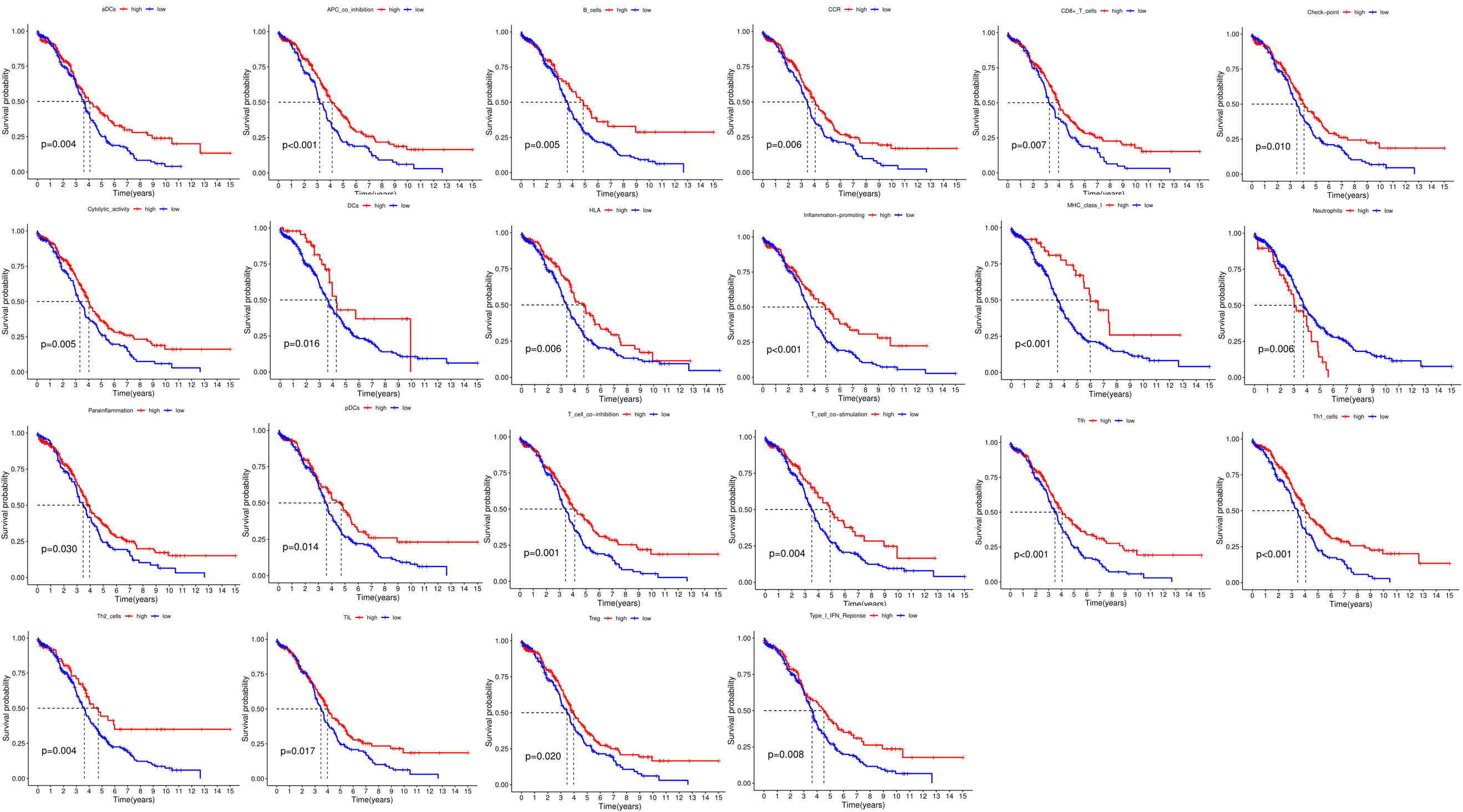

Supplement: Supplementary Figure 5 — Survival analysis of different abundance of the immune functions. [file Image_5.tif]

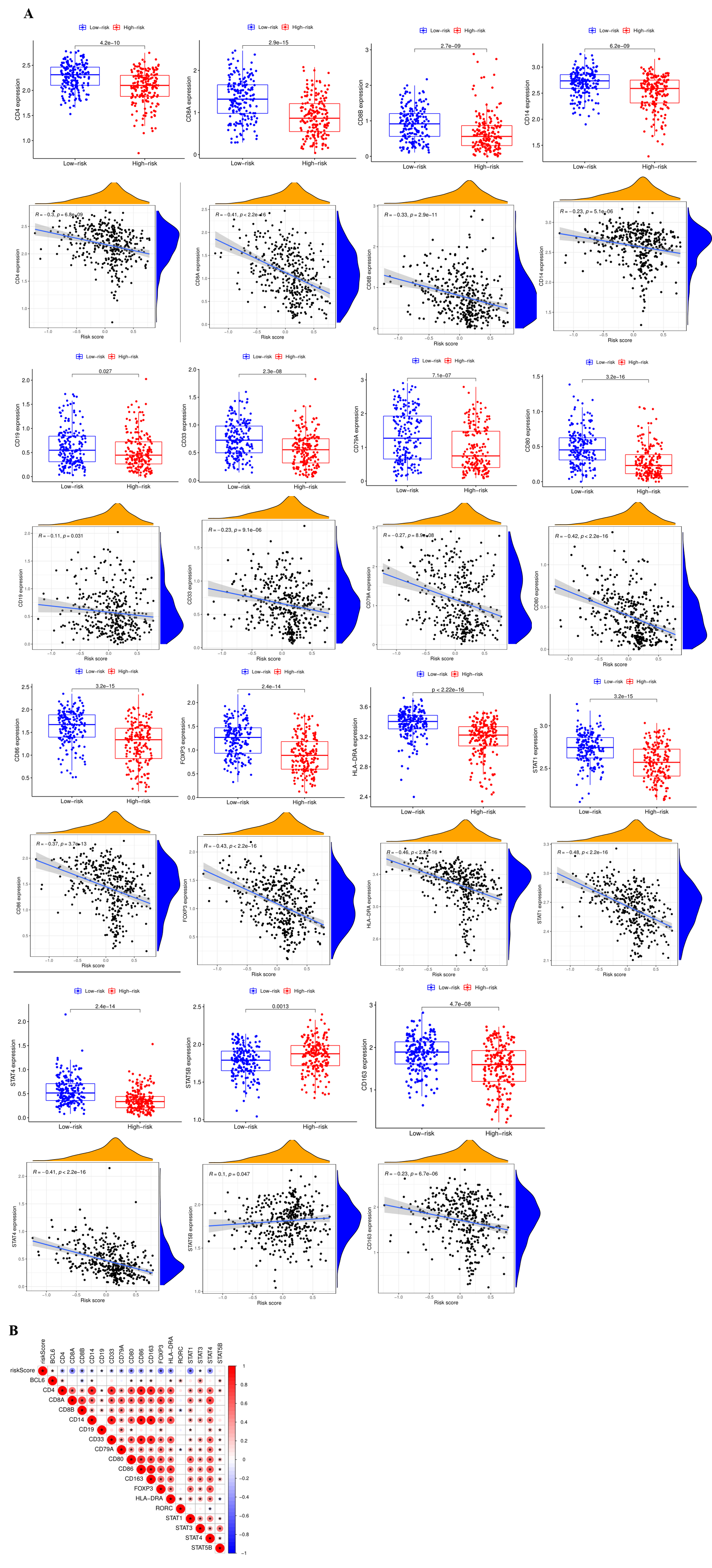

Supplement: Supplementary Figure 6 — Association between the model and immune cells (A) The expression profile of the immune cell markers and the correlation with the model between high and low risk groups (B) The association between the risk score and immune cell markers. [file Image_6.tif]

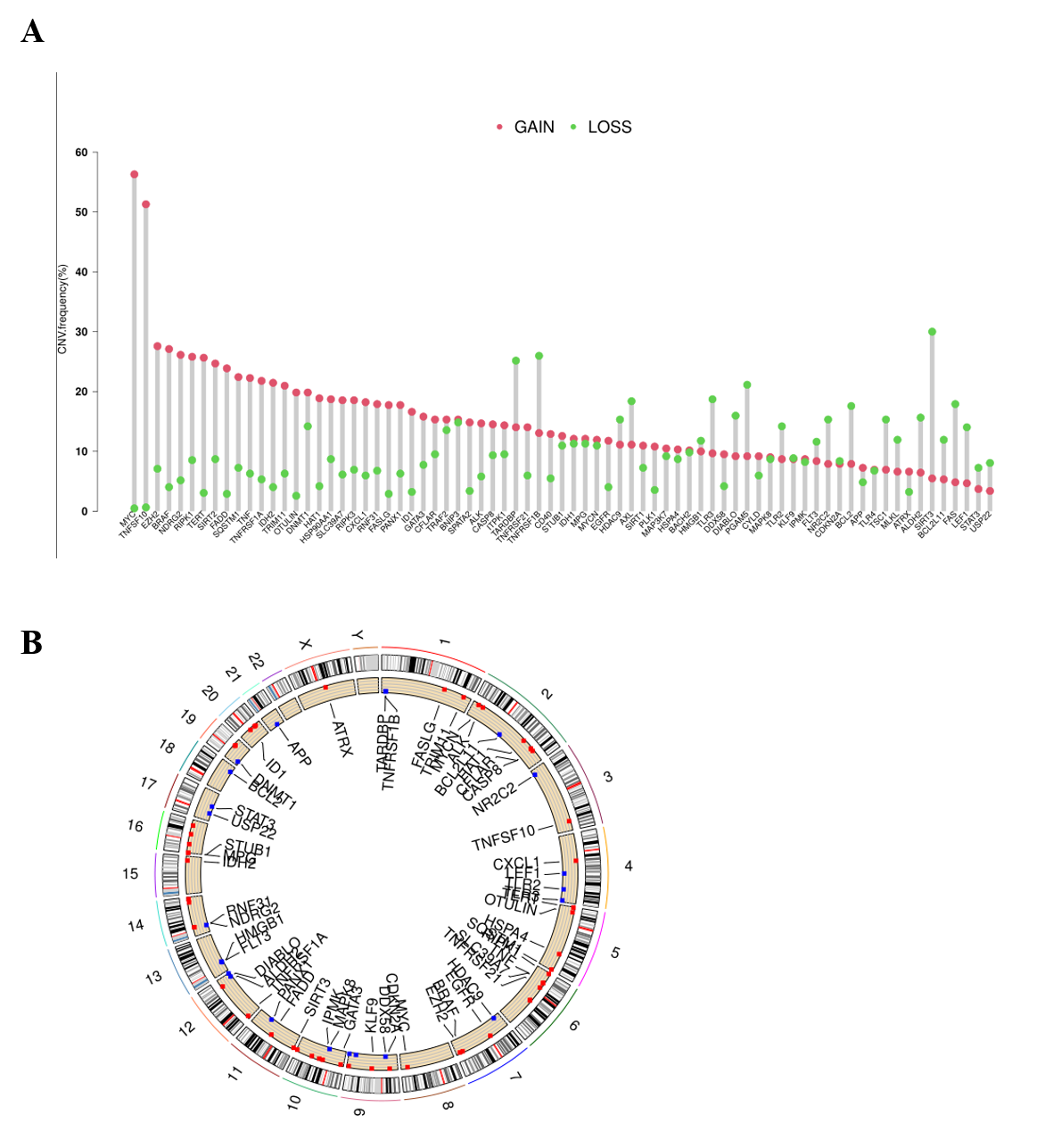

Supplement: Supplementary Figure 7 — CNV of the necroptosis-related genes in OC(A) CNV frequency of the necroptosis-related genes (B) Circos plot of the CNV spectrum. [file Image_7.tif]

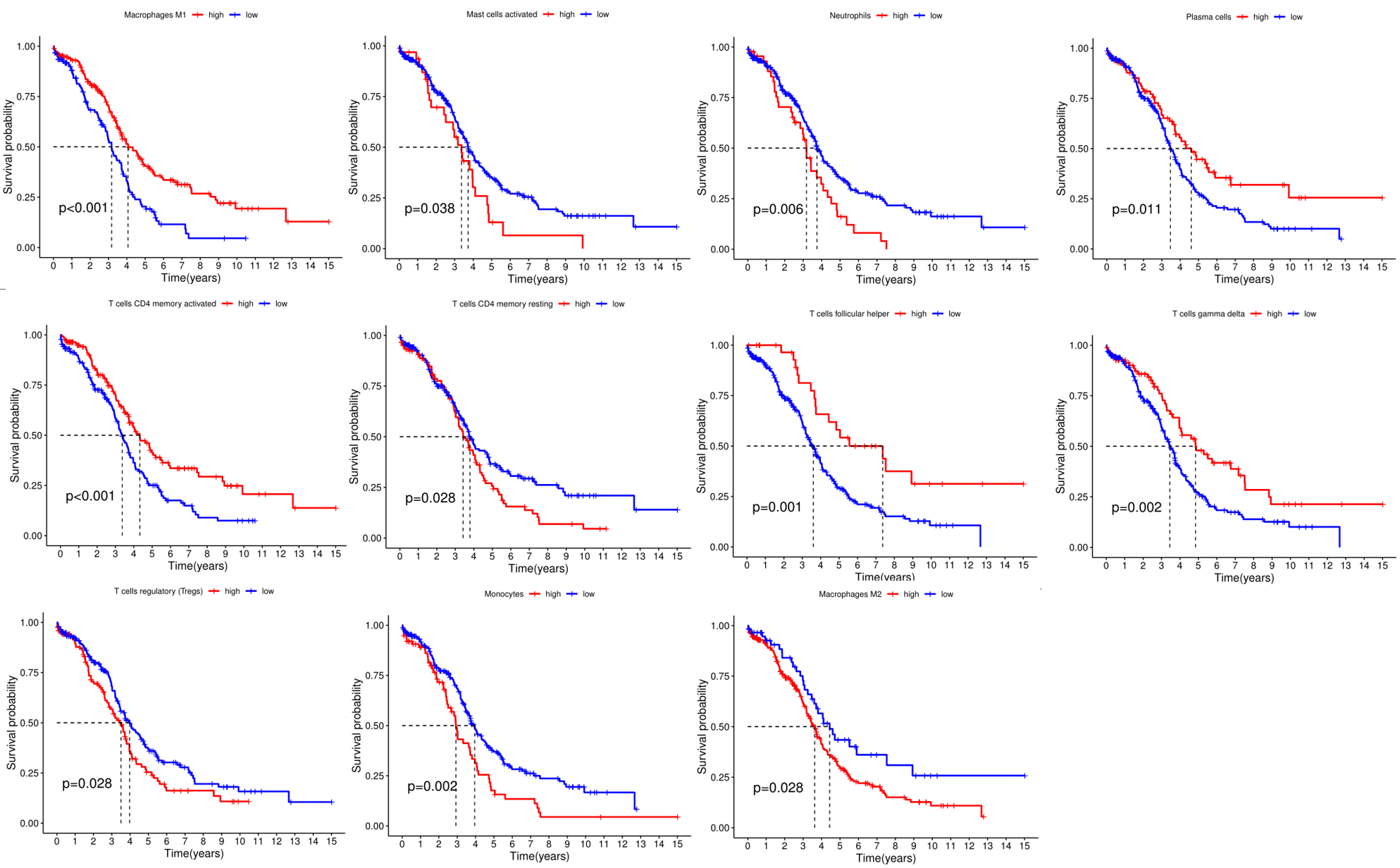

Supplement: Supplementary Figure 8 — Survival analysis between high and low degrees of immune cells. [file Image_8.tif]

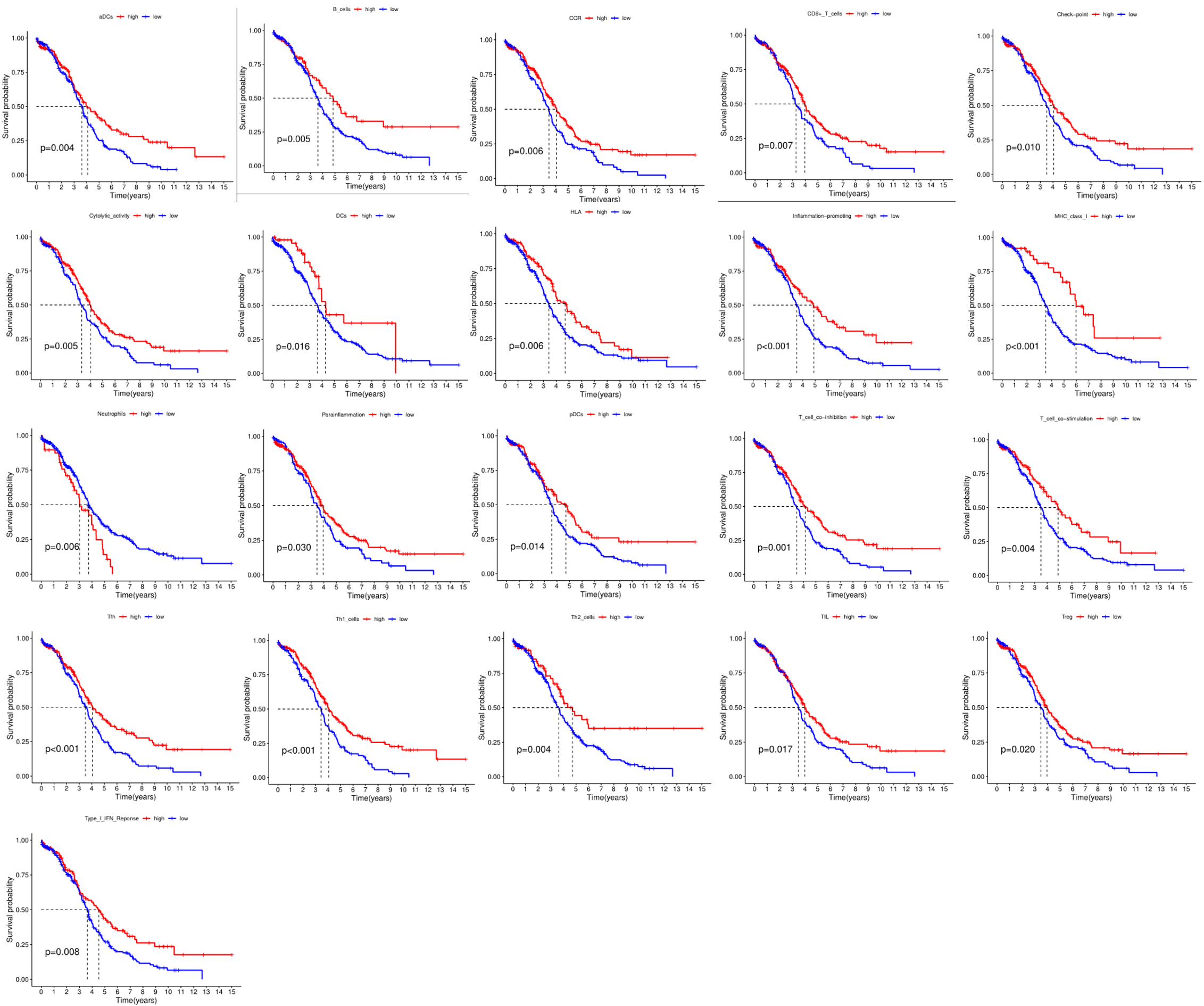

Supplement: Supplementary Figure 9 — Survival analysis between high and low degrees of immune functions. [file Image_9.tif]

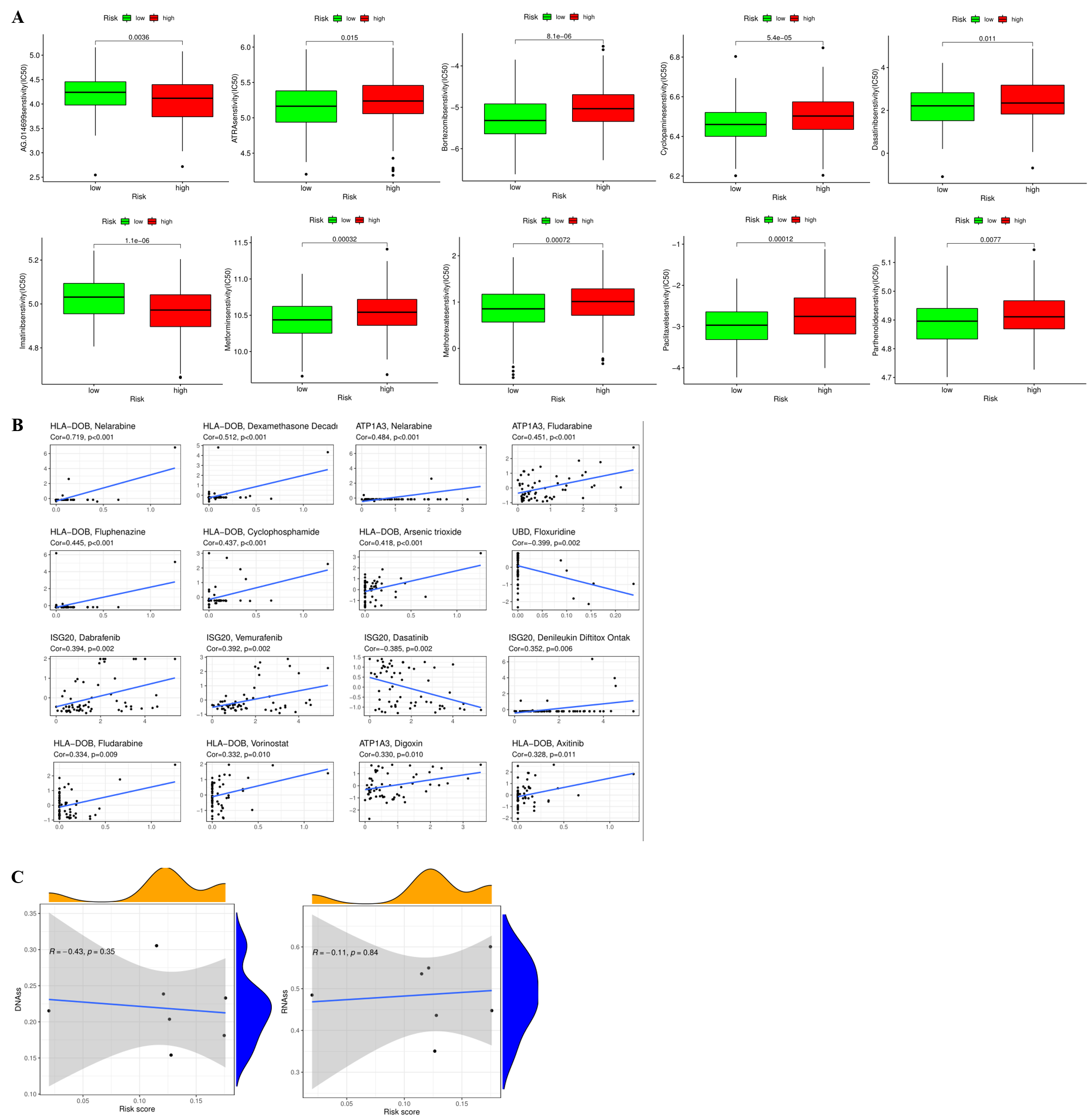

Supplement: Supplementary Figure 10 — Chemosensitivity and stemness of different groups (A) IC50 of multiple chemotherapy drugs between groups(B) Correlation between the risk genes and chemotherapy drugs (C) Assessment of the association of stemness and risk score. [file Image_10.tif]

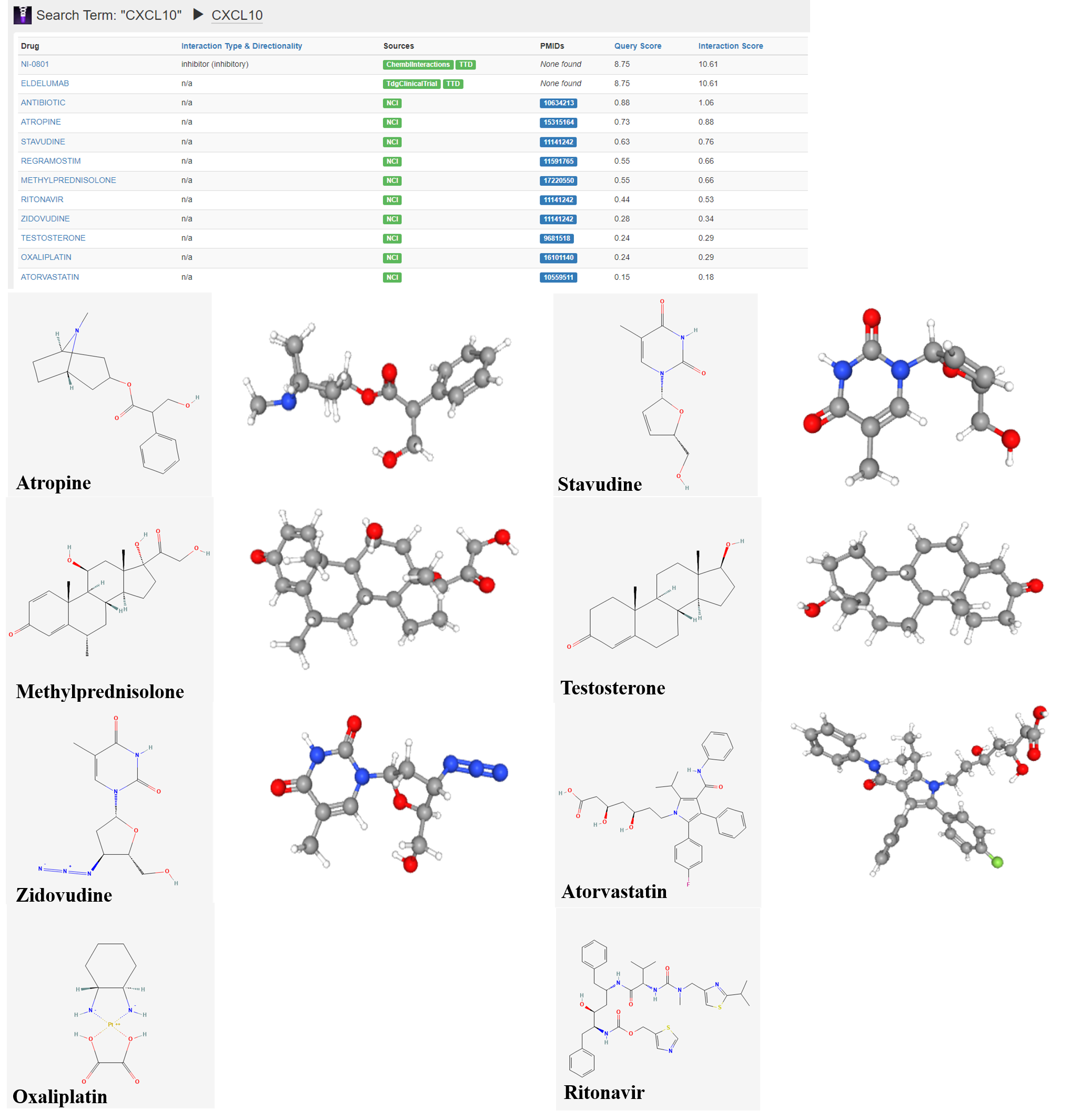

Supplement: Supplementary Figure 11 — The structure of predicted targeted drugs based on the DEGs in different groups. [file Image_11.tif]
